# Monoclonal Antibodies and Derivatives: Therapeutic Tools for Cancer

**DOI:** 10.32604/or.2026.078483

**Published:** 2026-06-16

**Authors:** Alessandro Poggi

**Affiliations:** Molecular Oncology and Angiogenesis Unit, Ospedale Policlinico San Martino, Genoa, Italy

**Keywords:** Antibody drug conjugates, tumor immunology, antibody-dependent cellular cytotoxicity, tumor microenvironment, innate immunity

## Abstract

The production of murine monoclonal antibodies (mAbs) with defined specificity in 1975 marked the subsequent revolution of cancer therapy. mAbs have been essential to characterize the functional features of molecules involved in cancer cell growth and dissemination. The murine mAbs have been modified to create humanized antibodies and, subsequently, fully human antibodies for cancer therapy, thereby avoiding the side effects of xenogenic protein. The antibody-drug conjugates (ADCs) increased the antitumor effect of mAbs. We will analyze the functional features of ADCs that recognize the cluster differentiation (CD)30 receptor present on some lymphomas and the human epidermal growth factor receptor (HER)2 on solid tumors. The anti-CD30 brentuximab vedotin and the anti-HER2 trastuzumab deruxtecan are two paradigmatic examples to understand the rationale of using mAbs against cancer. Some of the advantages, disadvantages, and clinical applications of these ADCs will be considered. The therapy with antibody derivatives, such as bispecific antibodies (BsAbs) and chimeric antigen receptor (CAR) cells for either CD30 or HER2, increased the potency and efficacy of specific targeting. Therapeutic antibodies directed to other members of the HER2 family, such as EGFR, or to immune checkpoint molecules, such as Programmed-death receptor (PD)-1 and PD-ligand (L) 1, will be analyzed for their ability to shape the tumor microenvironment (TME). Some mentions of functional features of mAbs linked to molecular glue degraders or stabilizers will be analyzed to highlight the potential evolution of ADCs to deliver undruggable molecules. As mimetics of antibodies, the affibodies will be briefly considered to give a more comprehensive scenario of the therapeutic tools that can target a molecule specifically. Overall, this review summarizes the evolution of antibody-based cancer therapeutics, focusing on the mechanistic underpinnings and clinical progress of ADCs, BsAbs, and CAR therapies, while highlighting emerging strategies to overcome resistance and modulate the TME.

## 1 Introduction

Classically, the therapy of cancer is based on interference with the proliferation of neoplastic cells using cytotoxic drugs and/or radiation. The main drawback of these treatments is the lack of cell target specificity, leading to the damage and killing of healthy cells. To overcome this problem, it is well-established that the key role of monoclonal antibodies (mAbs) and some of their derivatives, including humanized, fully human, single-chain variable fragments (scFv) of chimeric antigen receptors (CAR), bispecific (BsAb), bikes, trikes, antibody drug conjugates (ADCs), and radiolabeled antibodies [1]. This possibility was just an undefined hypothesis until, in 1975, Köhler and Milstein described the technique for the production of murine mAbs [2]. From the abstract of the original article, the authors stated, “We describe here the derivation of a number of tissue culture cell lines that secrete anti-sheep red blood cell (SRBC) antibodies. The cell lines are made by fusion of a mouse myeloma and mouse spleen cells from an immunized donor” [2]. The possibility of producing molecules binding with a specific antigen on a target cell opened a new era for the progress of basic research and clinical application of antibodies [3]. Early, murine mAbs represented a key tool for studying several basic biological processes, identifying new receptors involved in the activation/regulation of the immune functions, as well as molecules responsible for the growth and survival of cancer cells [1]. Afterward, the advancement of molecular biology engineering made possible the generation of chimeric and humanized mAbs useful to treat different hematological malignancies and solid tumors. At present, the market for antibodies is estimated to be worth around $20 billion, and these therapeutic tools have been applied in cancer therapy as well as for autoimmune diseases and transplantation [4,5,6,7,8].

The antitumor effect of the mAb can be achieved by the direct competition with the natural ligand for the growth factor receptor, as anti-EGFR mAbs [9,10,11,12,13] or influencing the heterodimerization of growth receptors, as for human epidermal growth factor receptor (HER)2 surface molecules [14,15,16,17,18]. Furthermore, mAbs can trigger the activation of the immune system, leading to the killing of tumor cells [15,16,17]. This activation involves mainly innate effector cells such as monocyte/macrophage (Mo/MΦ), polymorphonuclear (PMN) cells, and natural killer (NK) cells [18,19,20,21]. The damage to tumor cells induced by innate cells can lead to tumor target cell phagocytosis and killing [22,23,24,25,26], besides amplifying cytokine and chemokine cascades involved in the shaping of the tumor microenvironment (TME) [27,28,29,30,31] by activating the antibody-dependent cellular cytotoxicity (ADCC) and/or complement-dependent cytotoxicity (CDC) [32,33,34,35,36]. It is of note that some therapeutic mAbs have been modified to avoid both CDC [37,38,39], and ADCC [40,41,42]. Importantly, the targeting with mAbs can be directed not only to tumor cells but also to components of TME such as fibroblasts and immune cells, to trigger the anti-tumor host response.

In this review, we report some chemical and functional features of therapeutic mAbs and ADCs directed to paradigmatic examples of tumor targets such as cluster differentiation (CD)30 on lymphoma B cells or HER2 on breast carcinoma. Further, the most novel derivatives of mAbs, such as CAR cells or BsAb, generated to overcome the side-effects as well as the insurgence of resistant tumor cells [43,44,45,46,47] will be analyzed. Finally, the recent evolution of these tools to carry undruggable compounds, as well as their pitfalls, will be considered.

This approach aimed to give a general, but up-to-date, overview of the scenario for present and future clinical application of mAbs and derivatives.

## 2 ADCs: A Solution for Increasing the Efficacy of Chemotherapy

**Figure 1:**
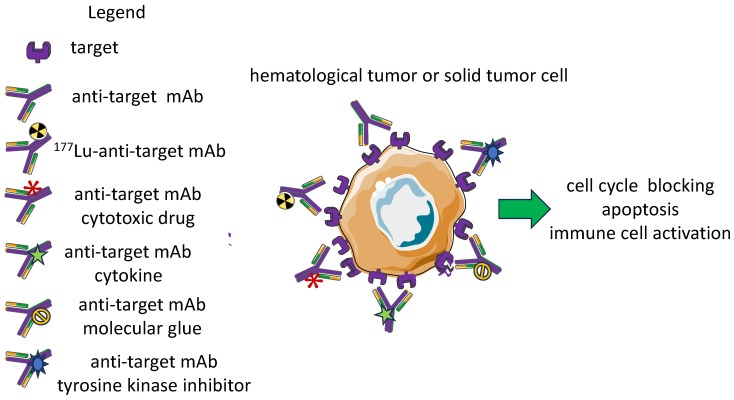
**Targeting tumor cells with monoclonal antibodies (mAbs) and some of their derivatives of tumor cells.** Both hematological malignancies and solid tumors can be targeted with mAbs. The target antigens are neo or overexpressed on tumor cells (depicted in purple). The mAbs can be used in the native form competing with the natural ligand of the target molecule, and interfering with the ligand-mediated signaling. Also, mAbs can be conjugated with several chemical compounds. The conjugation with radioactive isotopes is used both for imaging and therapeutic options. The presence of a cytotoxic drug as the payload restricts the cytotoxic effect to tumor target cells. mAbs are linked to immune-activating cytokines or molecular glue degraders or stabilizers or tyrosine kinase inhibitors. These payloads can stimulate the antitumor immunity or impair the cell cycle and stimulate apoptosis. This figure has been designed from the templates of Anatomy and Human Body and Cellular Biology series of Smart Servier Medical Art (https://smart.servier.com/).

Besides surgery and radiotherapy, chemotherapy is a key tool to eliminate cancer cells both for solid and hematological tumors [48,49,50,51]. One of the major drawbacks of chemotherapy is the toxic effect on healthy cells [52,53,54]. Indeed, some chemotherapy drugs target key biological molecules involved in the assembly of the cytoskeleton or on DNA, leading to impairment of cell growth [55,56,57,58]. Cancer cells are a good target because they are actively proliferating, and the inhibition of the expansion of tumor cells limits the progression and fatal outcome of cancer disease. The use of mAbs directed to specific surface molecules expressed mainly by tumor cells linked to the cytotoxic drug reduces the off-target side effects of the chemotherapy [53,59,60,61]. Also, the mAbs can be linked to growth/regulatory factors generating the immunocytokines, radioactive isotopes, chemical compounds targeting tyrosine kinases or other cellular enzymes, or molecular glues [60,62,63,64,65]. Fig. 1 gives a schematic representation of these immunoconjugates and the possible cellular targets applied in cancer research.

**Figure 2:**
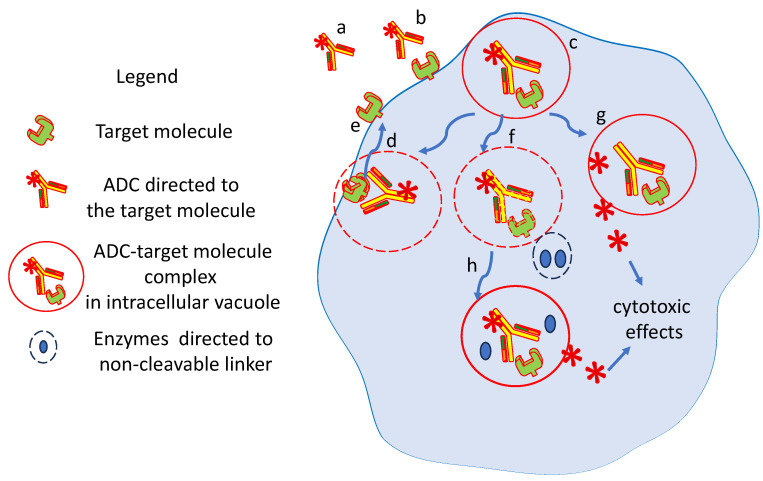
**Cellular processing of the ADC.** The antibody (a) directed to a specific tumor cell receptor (represented in green) interacts with this receptor, generating a complex (b). This complex is endocytosed (c). The antibody-receptor/antigen complex can undergo direct recycling (d), leading to re-expression of the receptor at the cell surface (e) or to the fusion with lysosomes (f), or other intracellular vesicles (g) in which the presence of enzymes (f) or acidic environment can induce the release of the drug payload (h). Also, the antibody-receptor/antigen complex can directly release the payload in the acidic vacuole microenvironment. The drug payload released can exert its cytotoxic effect, inducing programmed cell death of the target cell. Note: This general mechanism underlies the action of specific ADCs discussed herein, such as BV (anti-CD30; see Section 2.1) and T-DXd (anti-HER2; see Section 2.2). This figure has been designed from the templates of Anatomy and Human Body and Cellular Biology series of Smart Servier Medical Art (https://smart.servier.com/).

Typically, the ADC recognizes a tumor-associated antigen at the cell surface of a tumor cell. After antibody-antigen interaction, the complex is endocytosed by clathrin-coated vesicles or caveolae [66,67]. The endosomes undergo fusion with lysosomes, and in the presence of an acidic environment and/or by the action of intracellular degrading enzymes, the payload is released, and it can reach its molecular target. This latter interaction leads to the final cytotoxic effect, depending on the role of the target molecule (cytoskeletal protein, DNA). The tumor cell death is usually a programmed and complex process [68,69]. A brief and schematic description of how an ADC can enter a target cell is shown in Fig. 2.

**Figure 3:**
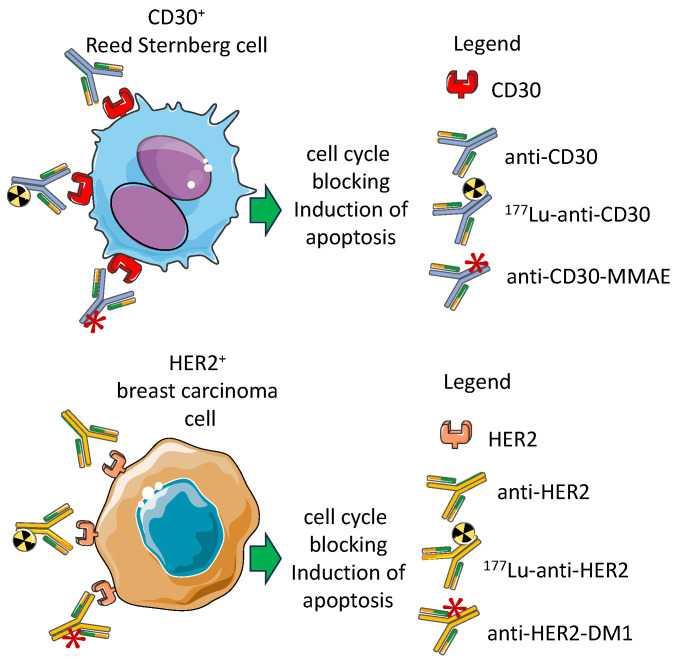
**Targeting of CD30^+^ hematological malignancies and HER2^+^ solid tumors with ADC.** The anti-CD30 antibody can interfere with this member of the tumor necrosis factor (TNF) receptor family. The use of the isotope-labeled antibody can lead to the cell cycle blocking and consequent apoptosis of CD30^+^ cells, as well as the anti-CD30 antibody linked to the tubulin inhibitor monomethyl auristatine E (MMAE). Similarly, the anti-HER2 antibody can impair the heterodimerization of the HER2 molecule with other components of the epidermal growth factor receptor (EGFR) family, inhibiting the consequent pro-proliferating signal. The anti-HER2 labeled with isotope or linked to the mertansine (DM1) drug specific for the cytoskeleton can trigger the killing of the solid tumor cell. This figure has been designed from the templates of Anatomy and Human Body and Cellular Biology series of Smart Servier Medical Art (https://smart.servier.com/).

This review does not cover all the mAbs and ADCs used as therapeutic tools that have been well-reviewed elsewhere [1,8,70,71]. Herein, we report some chemical, functional features, and clinical efficacy of ADCs targeting CD30 or HER2, two key surface molecules expressed on several lymphomas or carcinomas, respectively (Table 1 and Fig. 3). This choice is based on the evidence of the stronger efficacy of these mAbs to treat the target tumor than classical chemotherapy. Also, mAb derivatives such as BsAb and CAR cells specific to CD30 or HER2, besides other molecules, will be analyzed to reinforce the notion that these tools can be used to face resistant cancers. In the last part of the review, the most recent and innovative approaches to increase the anti-tumor immunity of mAbs, mAb derivatives, and mimetics of mAbs will be reported and discussed.

**Table 1:** Representative examples of antibody-drug conjugates (ADCs) targeting molecules in some hematological malignancies or solid tumors.

Antibody Drug Conjugate Name	Commercial Name/Phase Clinical Trial	Target Antigen	Chemical Linker	Drug Conjugated	Main Clinical Indication	Mechanism of Action
Brentuximab Vedotin	Adcetris	CD30	VC dipeptide	MMAE	HL ALCL	Inhibits microtubule polymerization, blocks mitosis
Fan Trastuzumab Deruxtecan-nxki	Enhertu DS-8201a	HER2	MC GGFG (Gly-Gly-Phe-Gly) tetrapeptide	DXd	HER2^+^ breast carcinoma	Inhibits DNA replication Topoisomerase I inhibitor DNA damage and apoptosis
Trastuzumab Emtansine	KadCyla	HER2	Non-cleavable SMMC	DM1	HER2^+^ breast carcinoma	Inhibits microtubule assembly
Trastuzumab duocarmazine	Jivadco SYD285 Phase III (NCT03262935)	HER2	cleavable	duocarmycin	HER2^+^ breast carcinoma	Alkylates adenine bases (N3 position) Blocks replication and transcription
Depatuxizumab Mafodotin	Phase III clinical trial	EGFR	Non-cleavable (MC)	MMAF	Glioblastoma Other EGFR^+^ tumors	Inhibits microtubule polymerization

Abbreviations: ALCL: Anaplastic Large Cell lymphoma; BC: Breast Carcinoma; CD: cluster differentiation; EGFR: Epidermal Growth Factor Receptor; HER2: Human Epidermal growth factor Receptor 2; HL: Hodgkin Lymphoma; MC: MaleimidoCaproyl linker; SMMC: Succinimidyl 4-(N-Maleimido Methyl) Cycloexane-1-carboxilate linker; MMAE: MonoMethyl Auristatine E; DXd: DeruXtecan; DM1: Emtansine; MMAF: MonoMethyl Auristatine F; VC: Valine-Citrulline linker.

### 2.1 Targeting of CD30 Antigen in Hodgkin Lymphoma and Other CD30^+^ Lymphomas

A typical example of ADC is represented by the anti-CD30 BV (commercial name Adcetris) [72] (Fig. 3 and Table 1).

This antibody recognizes the CD30 antigen expressed on activated B and T lymphocytes as well as some B-cell lymphomas, such as Hodgkin Lymphoma (HL) and anaplastic large cell lymphoma (ALCL), peripheral T-cell lymphoma (PTCL), mycosis fungoides (MF), adult T-cell leukemia/lymphoma (ATL), and a portion of diffuse large B-cell lymphoma (DLBCL) [73,74,75]. Also, CD30 can be expressed upon viral infection, such as human T-cell leukemia virus type 1 (HTLV-1); this virus can immortalize T cells and leads to carcinogenesis [76,77,78]. The targeting of CD30, a member of the tumor necrosis factor receptor superfamily (TNFRSF) [74], is based on the relevant role in the pro-survival signal through several biochemical pathways [73,74,75,76,77], including nuclear factor (NF) κB-mitogen-activated protein kinase (MAPK) activated upon the CD30 engagement by TNFSF8 (CD153) cytokine [78,79,80,81,82]. Importantly, CD30 can be shed by tumor cells upon the enzymatic activity of ADAM10 [83,84,85] and metalloproteinases [86,87,88]. The use of BV alone or in combination with chemotherapy and/or immune checkpoint inhibitors (ICI) is a treatment for relapsed/refractory (r/r) HL [89,90,91] and other lymphomas and leukemia [92,93,94,95]. This ADC is composed of a chimeric mouse anti-CD30 antibody (clone cAC10) conjugated with the microtubule inhibitor (monomethyl auristatin, MMAE) through a protease-cleavable linker that covalently attaches MMAE to cAC10 [93]. The intravenous administration leads to 100% drug absorption. The ADC interacting with the CD30 antigen on target cells undergoes endocytosis, and in lysosomes, the MMAE is cleaved from the antibody, and it alters the network of cytosolic microtubules, leading to impairment of cell division, and eventually, target cells die by apoptosis [95]. The conjugation to the antibody considerably limits the intrinsic toxicity of MMAE, increasing the antitumor-specific effects. It is of note that MMAE cannot be administered as itself, and the toxicity is clearly different when administered as a payload of an ADC, and the toxicity of ADC rarely depends on the antibody targeting but mainly on the MMAE payload released from the antibody [94,95,96,97].

Recently, the anti-CD30 antibody cAC10 has been coupled with the radiometal chelator DOTA, with the ^177^lutetium or the ^161^terbium generating the Lu-cAC10 and Tb-cAC10 radiolabeled antibodies [98]. These conjugates possess high specific activities, radiochemical purity, and strong plasma stability, with more than 90% of the radioactivity associated with the antibody also after 7 days from inoculation in mice. The cytotoxic effect on some CD30^+^ ALCL cell lines, such as Karpas 299 and Mac2A, and the cutaneous T cell lymphoma cell line Myla, was proportional to the level of expression of the CD30 receptor. *In vitro*, the two radiolabeled ADCs led to the killing of cells through the induction of double-strand breaks. The assessment of therapeutic efficacy was analyzed *in vivo* with a subcutaneous Karpas 299 xenograft model in athymic nude mice. A single administration of the ^161^Tb-cAC10 led to a better survival time (45 days, and 4 mice did not die) of mice compared with ^177^Lu-cAC10 (35 days), although the latter could prolong the life of tumor-bearing mice to 35 days from 25 days (untreated mice). The proteomic and phosphor-proteomic analysis indicated that both the ADCs affect pathways regulating response to DNA damage and the progression of the cell cycle. These findings would suggest that radioimmunotherapy with ^161^Tb-cAC10 could be a good alternative to overcome the side effects of BV, such as cumulative peripheral neuropathy [99]. Furthermore, although the BV showed effectiveness against HL, the therapy of r/r HL and other CD30^+^ lymphomas is challenging due to the high rate of relapses in aggressive types such as ALCL [100]. The therapy is affected by the generation of resistant tumor cells [96,97,100,101,102]. Indeed, some patients develop intolerance of BV therapy in combination with ICI, such as anti-PD1, or simply become insensitive to BV [102]. Furthermore, the radiolabeled antibodies versus CD30 antigen can be used for theranostic purposes [103]. The anti-CD30 antibody IMB16 has been labelled with either ^64^Cu or ^177^Lu and used for immuno-positron emission tomography (immuno-PET) and radioimmunotherapy (RIT) in a subcutaneous NSG murine model. Results indicated that the [^64^Cu] Cu-NOTA-IMB16 enabled non-invasive assessment of CD30 expression, while the [^177^Lu] Lu-DOTA-IMB16 could efficiently affect the growth of CD30^+^ cells *in vivo* [103]. Overall, radiolabeled anti-CD30 antibodies could be a future therapeutic tool for some lymphomas, possibly in association with other anti-lymphoma drugs or biologicals.

### 2.2 Breast Carcinomas HER2^+^ Are a Paradigmatic Target for ADC in Solid Tumors

A second paradigmatic example of ADC is represented by the anti-HER2 antibody trastuzumab (Fig. 3 and Table 1) linked to deruxtecan or emtansine [104,105]. This ADC has been used for the treatment of overexpressing HER2^+^ breast carcinomas (BC), showing a better efficacy of the unconjugated antibody [106,107,108]. The antibody trastuzumab only led to an improvement of disease-free survival (DFS) among women compared to simple observation after excision of early-stage breast cancer and completion of chemotherapy [106] (ClinicalTrials.gov number, NCT00045032 phase III). Trastuzumab antibody was approved by the Food and Drug Administration (FDA) for the treatment of metastatic (m) BC in 1998. Unfortunately, the majority of patients develop resistance to trastuzumab treatment within one year after initial treatment, suggesting the activation of anti-HER2 antibody molecular mechanisms leading to relapse of the tumor [108,109,110]. The resistance to anti-HER2 therapy is linked to the strong heterogeneity of expression of HER2 antigen among tumor cells and to the type of test applied to define HER2^+^ overexpressing BC [111,112]. Indeed, the strong expression of HER2, indicated as 3+ in immunohistochemistry (IHC), should be present on more than 10% of tumor cells to classify the BC as a HER2 overexpressing BC. This observation suggests that a large portion of BC cells can express lower amounts of HER2 than those identified by a 3+ staining. Thus, it is conceivable that HER2-low tumor cells can escape from the targeting with anti-HER2 antibodies and ADC [110,113,114].

Recently, it has been shown that the abnormal processing and transport of the anti-HER2 ADC can affect the antitumor effect against BC [115]. In this case, the vesicle transport-related gene, sorting nexin 10 (SNX10), shows a key role in the correct transport of the anti-HER2 ADC. This suggests that not only the large heterogeneity of expression of the HER2 antigen but also intracellular transporters are involved in the failure of antibody- or ADC-mediated BC therapy. The role of SNX10 was identified in patient-derived organoids (PDO) and in the cell line SKBR3 selected from the parental cell line (SKBR3) as resistant *in vitro* to trastuzumab treatment (SKBR3-TR). Indeed, the SNX10 was markedly downregulated in SKBR3-TR, and its downregulation in the parental SKBR3 cells or its upregulation in SKBR3-TR cells either led to a reduced or an increased sensitivity to treatment with T-DXd or T-DM1, respectively. Importantly, the reduction of SNX10 increased the HER2 protein degradation, while the upregulation of SNX10 led to the opposite effect. The two main cellular protein degraders are represented by the ubiquitin-proteasome system and the autophagy-lysosome system that show a relevant cross-talk [116,117] and have been tested for their role in processing the HER2 molecules [115] using inhibitors of one or another system. The use of lysosome inhibitors could restore SNX10 deficiency–induced HER2 degradation. Furthermore, the SNX10 regulates the HER2 cell surface expression as well as the RAB11A endosomal expression. Altogether, these findings suggest that SNX10 is a key molecule regulating the expression and recycling of the HER2 [115].

An additional example of an anti-HER2 ADC is the trastuzumab-duocarmazine (Table 1, named SYD985) [118,119,120]. This ADC bears a cleavable linker and the alkylating agent duocarmycin that targets the minor groove of the DNA at A-T-rich sequences, leading to irreversible covalent binding to the N3 nitrogen of adenine. This linkage produces a distortion of the helix and consequent alteration in DNA replication and translation, blocking cell proliferation and protein synthesis. The attempts of DNA-repair mechanisms to solve this damage become quickly overwhelming, and the cells undergo cell suicide [121]. The SYD985 was more potent than T-DM1 in a mixed population of cells expressing different ranges of HER2. Furthermore, this ADC was efficient in eliminating low HER2-expressing cells in preclinical experiments, and a phase III study is ongoing (NCT03262935 Tulip trial) in BC progressing after the treatment with T-DM1. Importantly, the SYD985 is not yet approved by the FDA, and it was withdrawn by the European Medicines Agency (EMA) due to relevant concerns regarding the data analysis and patients’ follow-up, leading to uncertain benefits against risks. Indeed, the TULIP trial showed promising results in HER2^+^ BC with progression during/after ≥2 HER2-targeted therapies or after T-DM1 [122] with a better progression-free survival (PFS) than other treatments. However, a notable ocular toxicity accompanied the strong anti-tumor effects [122,123,124]. Indeed, in the phase I clinical trial study NCT02277717, most patients (104 of 146) had at least one ocular adverse event, with grade 3 events reported in ten of 146 patients. These findings indicate that anti-HER2 drug conjugates are clearly specific and potent anti-BC therapeutics, but the safety profile should be analyzed in detail, as the off-target effects can be severe and may lead to discontinuation of the therapy.

Also, the anti-HER2 radiolabeled antibodies, ^177^Lu-DO3A ADC, have been used to demonstrate the *in vivo* treatment efficacy compared to anti-HER2 MMAE conjugates. Importantly, the ^177^Lu-DO3A ADC showed better results against refractory tumors displaying a heterogeneous expression of the HER2 molecule [125]. More importantly, it has been recently reported that the use of ^89^Zr-radiolabeled anti-HER2 antibodies (trastuzumab or pertuzumab) can detect both HER2-strongly positive and HER2-dull positive BC cells. Interestingly, the previously identified cases as false positives were classified as HER2 low with anti-HER2 radiolabeled antibodies [125]. The implications for patients could be that HER2 radiolabeled antibodies can assess the presence of disease, although BC cells express low levels of HER2, characterizing better than IHC the follow-up and evolution of the BC. It may be hypothesized that anti-HER2 antibody radiolabeled with appropriate isotopes can target and eliminate BC cells independently of the level of expression of the HER2 molecule.

## 3 BsAb and CAR Immune Cells as Derivatives of mAbs for Cancer Therapy

The ADCs represent a great therapeutic advancement to target specifically tumor cells with potent cytotoxic drugs, but several pitfalls should be considered in failing the efficacy to eliminate tumor cells. Indeed, off-target cytotoxicity, dose-limiting adverse effects, inefficient tumor delivery, acquired resistance, antigen sink and shedding, as well as constitutive and acquired resistance are several problems that should be carefully considered [126]. To overcome some of these matters, therapeutic tools such as BsAb [127], and CAR cells have been generated [128,129,130,131,132] to kill tumor targets by activating the host anti-tumor immune response.

**Figure 4:**
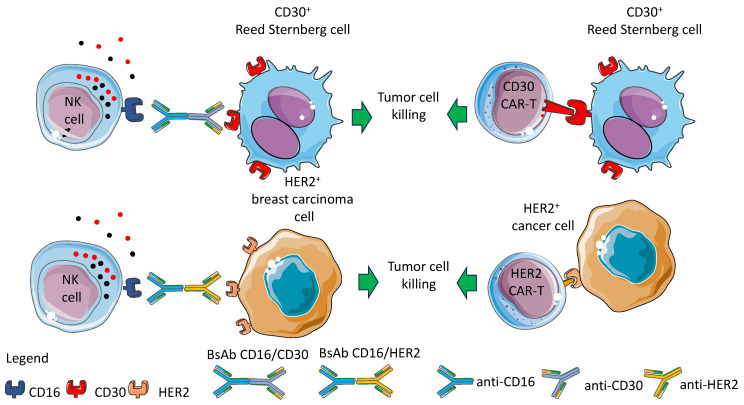
**Schematic representation of the targeting of CD30^+^ hematological malignancies or HER2^+^ solid tumors with bispecific antibodies or chimeric antigen receptor (CAR) cells.** Bispecific antibodies (BsAb) make a bridge between the tumor cell and the immune effector cell. The specificity of the antibody can recognize an activating molecule on immune cells, such as the CD16/FcγRIIIa, typically present on natural killer (NK) cells. The CAR-T cells are engineered cells with a chimeric molecule that can interact with the target antigen and deliver a triggering signal into the immune cell, leading to killing of the target and/or the release of antitumor cytokines. In the scheme are shown BsAb and CAR-T cells specific for either CD30^+^ Hodgkin lymphoma (HL) Reed-Sternberg cells or HER2^+^ tumor cells. Usually, these therapeutic tools are used in relapsed/refractory tumors previously treated with ADC and chemotherapeutic drugs. This figure has been designed from the templates of Anatomy and Human Body and Cellular Biology series of Smart Servier Medical Art (https://smart.servier.com/).

They represent the evolution of the conventional mAbs after essential modifications and engineering. Roughly, BsAbs show variable regions of two different mAbs, while the single-chain variable fragment (scFv) of an antibody is engineered with other components to become a new surface molecule able to transduce a signal. The CAR can be transduced into an immune effector such as αβ^+^T or γδ^+^T lymphocytes or NK cells or monocytes, and these effectors will eliminate the tumor targets [133,134,135,136,137]. Herein, we will analyze some of the BsAb and CAR-immune cells used for cancer therapy (Fig. 4).

### 3.1 BsAb to Target Hematological Malignancies or Solid Tumors

Typically, mAbs can recognize one specific antigen/epitope, while BsAb can recognize two distinct specific antigens/epitopes at the same time. Usually, the BsAb can bridge a tumor target cell with one specificity and an immune cell with the second specificity. This leads to the targeting of that tumor target cell by the immune cell, favoring the binding of the effector immune cell to the tumor; the consequent activation of the immune cell, if the specificity of one arm of BsAb is an activating receptor, can trigger the killing of the target cell [127,138,139,140,141]. Table 2 summarizes some of the BsAbs that are under investigation, directed to different target molecules. It is evident that these BsAb engage immune molecules such as CD3 or FcγR, inducing a strong antitumor immune response. It is of note that several ongoing clinical trials are in the first phases of the clinical application of these tools. Indeed, some major matters should be considered when targeting CD3 and FcγR, such as cytokine release syndrome (CNS), neurological involvement (ICANS), and on-target off-tumor cytotoxicity due to the indiscriminate triggering of potent immune effector cells such as T and NK cells able to produce proinflammatory cytokines such as IFNγ and TNFα. Besides the generation of resistance favored by the heterogeneity of tumor cells and TME immunosuppression, the manufacture/development of BsAb is a challenge, and the costs can markedly increase [142].

**Table 2:** Representative examples of BsAb directed to some tumor target molecules, such as CD30 or HER2, in some hematological malignancies or solid tumors.

Bispecific Antibodies Name	Stage of Developing/Clinical Trial/Phase	Target Antigens	Functional Effect	Immune Cell Triggered	Main Clinical Indication
AFM13	NCT02321592/II NCT03192202/Ib-IIa NCT02665650/I NCT04074746/II NCT05883449/II NCT04101331/II NCT01221571/I	CD30 × CD16A	Killing CD30^+^ lymphoma cells	NK cells NK-T cells	r/r HL PTCL NHL ALCL
8D10 10C2 AC10 x OKT3	preclinical	CD30 × CD3	Killing CD30^+^ lymphoma cells	T cells	SU-DHL-1, Raji LV30, RPMI 6666 cell lines
Zenocutuzumab (MCLA-128)	NCT02912949/I-II	HER2 × HER3	reduce cell proliferation, and survival	ADCC	Solid tumor NRG1 Fusion protein NSCLC, PDAC
Zenocutuzumab (MCLA-128)	NCT04100694/I-II	HER2 × HER3	reduce cell proliferation, and survival	ADCC	NRG1 fusion PDAC NSCLC
Zenocutuzumab (MCLA-128)^+^ trastuzumab/chemotherapy	NCT03321981/II	HER2 × HER3	reduce cell proliferation, and survival	ADCC	mBC ER^+^ and low HER2
Zenocutuzumab (MCLA-128)	NCT05588609/II	HER2 × HER3	reduce cell proliferation, and survival	ADCC	NSCLC Castration resistant m PC
MDX-447	NCT00005813/I	EGFR × FCγRI	EGFR^+^ cells	LAK cells	GBM Brain tumors
CM336	NCT07115654/II	BCMA × CD3	Killing BCMA^+^ cells	T cells	POEMS syndrome
CM336	NCT06745687/I	BCMA × CD3	killing BCMA^+^ cells	T cells	SM MM
3F8xOKT3	NCT03860207/I-II	GD2 × CD3	killing GD2^+^ cells	T cells	Osteosarcoma Neuroblastoma
KN046	NCT06834399/IIa	PDL1 × CTLA4	Relieving the brake of effector cells	T cells	CRC with liver metastasis
QL1706	NCT06404463/II	PD1 × CTLA4	Relieving the brake of Effector cells	T cells mainly	BC Early BC

ADCC: antibody-dependent cellular cytotoxicity; BCMA: B Cell Maturation Antigen; mBC. Metastatic Breast Carcinoma; CRC: ColoRectal Carcinoma; CTLA4: Cytotoxic T-Lymphocyte Antigen 4; NHL: Non-Hodgkin Lymphoma; r/r: relapsed/refractory; GBM: Glioblastoma Multiforme; GD2: disialoganglioside 2; ER: estrogen receptor; LAK: Lymphokine Activated Killer cells; NSCLC: Non-Small Cell Lung Carcinoma; MM: Multiple Myeloma; NK: natural killer; PDAC: Pancreatic Ductal AdenoCarcinoma; PTCL: peripheral T-cell lymphoma; PD1: Programmed cell Death protein 1; PC: Prostate Carcinoma; POEMS: Polyneuropathy Organomegaly Endocrinopathy Monoclonal gammopathy Skin alterations; SM: smouldering Myeloma.

Herein, we focus on BsAb targeting CD30^+^ lymphomas [139,140,141,142,143] or HER2^+^ BC [144,145,146,147,148], representing examples of the use of these antibody derivatives to face r/r hematological malignancies [149,150,151,152] or advanced stages of solid tumors [153,154,155,156,157]. It is of note that BsAb can be administered in combo therapies, such as with ICI, to improve their efficacy and counteract the immunosuppression of the TME, or BsAb can target IC molecules [158,159].

#### 3.1.1 Targeting CD30 with BsAb

A typical example of targeting CD30^+^ tumor cells is represented by the acimtamig (AFM13), a CD30/CD16A-bispecific antibody [160,161,162,163,164]. AFM13 is an innate immune cell engager as it makes a bridge between the CD30^+^ cells and the FcγRIIIA/CD16 expressed mainly on NK cells, besides some subsets of αβT cells and a large proportion of γδT cells [165,166,167]. The binding to the CD16 activating receptor triggers a strong activating signal in the immune cells, leading to the killing of the CD30^+^ cells and release of pro-inflammatory molecules such as IFNγ and TNFα [165,166,167,168]. Innate effector cells can kill the target through several means, such as perforins and granzymes (in a short time), and release or expression of FASL [169]. Importantly, the effect is linked to the recognition of the anti-CD16 expressed on the NK cell by the fragment antigen binding (Fab), not by the Fc portion of the antibody, as it happens in the classical ADCC triggered by therapeutic humanized/human antibody with the appropriate isotype of Fc, like the anti-EGFR cetuximab or the anti-HER2 trastuzumab [170,171]. The final result is always the activation of the cytolytic machinery of the effector cells.

In the phase II clinical trial NCT04101331, AFM13 has been administered in patients with r/r PTCL once a week for 8 cycles in 108 patients. The overall response (OR) rate was 32.4% with a complete response (CR) rate of 10.2 with a median duration of response of 2.3 months. Apparently, the clinical response was independent of the CD30 expression level, BV treatment, or steroid premedication. Although the treatment led to promising clinical activity, the OR by fluorodeoxyglucose-positron emission tomography (PET) was not reached. The tolerability was good, as the adverse effects related to the treatment were not strong, and no cases of cytokine release syndrome (CRS) or death have been detected [160]. A phase I clinical trial (NCT04074746) studied the response in patients with r/r CD30^+^ lymphoma resistant to BV and ICI [161] to cord blood cytokine-activated and activated NK cells precomplexed with AFM13. The persistence of NK cells reached up to 3 weeks, and trafficking at the tumor site could be detected. Importantly, no neurotoxicity, graft-versus-host disease (GVHD), or CRS have been reported; the OR and CR were 92.9% and 66.7%, respectively. The overall survival (OS) rate was 76.2% at a median follow-up of 20 months [163]. Also, the AFM13 BsAb administration with the anti-PD1 antibody pembrolizumab in the phase 1b clinical trial NCT02665650 has been tested in r/r HL patients [163]. This combo treatment was well tolerated with a similar safety profile to the two drugs alone. The objective response rate was 88%, with an OR rate of 83%. Altogether, these findings suggest that the use of the BsAb AMF13 can be effective alone or in combination in patients heavily treated and resistant to several conventional drugs.

Recently, it has been derived from AFM13 a novel tetravalent BsAb with silenced human IgG1 Fc (L234F/L235E/D265A). This modification aims to avoid the link of the BsAb to CD16b and to eliminate the potential differences in binding to NK cells expressing different polymorphisms of CD16. Also, this BsAb showed pharmacokinetics superior to that of AFM13 as well as the activation of ADCC [172]. It is of note that the modular design of this BsAb with the anti-CD30 component at the N-terminal of Fc can be modulated to other Fab specificities or variable heavy chains. This feature can make this BsAb a base for future developments and therapy of HL.

Regarding BsAb targeting the CD30 molecule with one arm, it is worth mentioning those with anti-CD3 or CD28 as the second arm [141,173,174]. Apparently, the effectiveness of targeting T cells instead of NK cells was higher, leading to the cure of all the SCID mice bearing HL tumors. Interestingly, the administration of both the CD30/CD3 and CD30/CD28 BsAb induced the activation of tumor-infiltrating lymphocytes (TIL) present in HL. These TIL expressed high levels of perforins and granzyme A and B. Also, the blocking of LFA1/ICAM1 or CD2/LFA3 interactions reduced the cytotoxicity mediated by BsAb; apparently, this inhibition was not due to the impairment of binding between the effector CD8^+^ T cells and the tumor target cells [175]. This observation suggests that adhesion molecules can play a key role in the effectiveness of CD30-targeted therapy. Also, these BsAb used for the treatment of SCID mice with HL tumors disseminated throughout the animal resulted in the complete cure [175]. This strong effect was also evident after 2 weeks from the initial inoculation of tumor cells, strongly suggesting the possible use of this combo of BsAb in r/r HL. Recently, the generation of a BsAb CD30/CD137 that targets RS cells and can trigger the ADCC of HL cells has been described, representing a good candidate for the possible conjugation with cytotoxic drugs and consequent killing of HL cells showing resistance to anti-CD30 antibodies [176].

#### 3.1.2 HER2^+^ Tumors As a Suitable Target for BsAb

HER2, as a member of the EGFR family, becomes activated after the homo- or heterodimerization with other components of the HER2 family, such as the EGFR or HER3 [177]. Its overactivity, usually achieved in cells overexpressing the HER2, is associated with unfavorable clinical outcomes [178]. The *in vitro* cytotoxicity of HER2/CD3 BsAb composed of the specificity of trastuzumab antibody, except for the N297A mutation in the Fc region, and the anti-CD3 mAb OKT3 correlated with the level of expression of HER2 on a wide panel of tumor cell lines (n = 39) [179]. Effector cells were represented by T cells from PBMCs stimulated with a combination of anti-CD3 and anti-CD28 mAbs for two weeks, preincubated with the BsAb, washing away the excess of the BsAb. These activated T cells were incubated with target cells, and killing was evaluated in a short-time cytotoxic assay. The EC50 ranged from 0.3 pM of the AU565 BC cell line (HER2 strongly positive) to >5000 pM of NCI-H345 small cell lung cancer (SCLC, HER2 very low). Some of the cell lines tested appeared to be insensitive to the treatment *in vitro* with some tyrosine kinase inhibitors such as erlotinib, lapatinib, or neratinib, or anti-EGFR antibody cetuximab or anti-HER2 antibody trastuzumab. However, the same cell lines were efficiently killed by activated T cells preincubated with the HER/CD3-directed BsAb. In addition, the cytotoxicity was partly independent of the expression of PDL1 on target cells or of PD1 on effector cells. Finally, this BsAb was effective against HER2^+^ humanized mouse models of BC and ovarian cancer cell line xenografts, as well as human BC and gastric cancer patient-derived xenografts (PDX) [179]. Altogether, these findings suggest that activated T cells can efficiently eliminate HER2^+^ tumor cells, also resistant to other HER2-targeted therapies, of different origins through the engagement of CD3 on effector cells and HER2 on target cells. This further supports the possible use of BsAb to treat resistant HER2^+^ cancers.

Importantly, HER2 is expressed on several healthy tissues besides tumor cells [180,181]. This leads to the possibility of on-target off-tumor toxicity, which can limit the clinical application of therapeutic means that trigger T cell-mediated killing of target cells, such as HER2/CD3 BsAb. The degree of affinity of both anti-CD3 and anti-HER2 components of the BsAb for the specific antigens is relevant for having an optimal balance between the tolerability and the efficacy of the treatment. Indeed, it has been shown that three different HER2/CD3 T cell-dependent BsAbs (termed TBD1, TBD2, and TBD3) have a specific different affinity for either CD3 or HER2. It is of note that lower CD3 affinity was better tolerated by non-human primates compared to a higher CD3 affinity. On the other hand, the higher HER2 affinity was associated with cytokine release syndrome in non-human primates, although the tumor cell killing was optimal. The CD3 affinity did not affect the sensitivity of *in vitro* killing of some cell lines, as well as the *in vivo* anti-tumor effect in mouse and non-human primate models. Importantly, the tolerability of HER2/CD3 BsAb was improved by fractionating the administered dose. These findings indicate that it is necessary to tune the affinity of the different arms of the HER2/CD3 BsAb to obtain an optimal balance between the anti-tumor effects, limiting the side effects due to HER2 expression on healthy cells, and the excessive activation of the T cell response.

BsAb can detect the two target antigens on the same cell, and the activation of the immune response can be triggered through the activation of the FcγR. This is the case of the IgG1 BsAb Zenocutuzumab (Zeno; MCLA-128), composed of HER2xHER3-directed antibody against tumor with neuregulin 1 (NRG1) gene fusion, which has been recently approved in the USA for metastatic and unresectable pancreatic adenocarcinoma or non-small cell lung cancer (NSCLC) [182]. NRG1 binds to HER3, causing heterodimerization with HER/ERBB kinases, stimulating cancer cell growth [183]. NRG1 fusion proteins are rare (<1% of solid tumors), but patients with NRG1^+^ tumors have limited therapeutic options due to the aggressive behavior of these cancers [184]. Zenocutuzumab prevents the heterodimerization of HER2 with HER3, impairing signal transduction mediated by NRG1, consisting of the PI3K-AKT-mTOR signaling leading to proliferation and promoting tumor cell survival [185]. The use of this BsAb can inhibit the phosphorylation of HER3 and AKT, trigger apoptosis, and inhibit cell growth of patient-derived cell lines and xenograft tumors in animal models. Also, Zenocutuzumab can trigger a potent ADCC signal in effector FcγR^+^ cells due to the engineered modified IgG1 subunit [185]. Importantly, two patients suffering from pancreatic ductal adenocarcinoma (PDAC) responded rapidly and maintained a radiographic and biomarker response for over 12 months [182,183,184]. It should be noted that in HER2^+^ BC, the targeting of the HER2/HER3 signaling axis may overcome ligand-mediated resistance to trastuzumab treatment [186]. This effect has been demonstrated using a triple combination of antibodies: trastuzumab (anti-HER2), pertuzumab (anti-HER2), and patritumab (anti-HER3). Altogether, these findings suggest that the signal through HER2/HER3 is relevant for cancer cell growth, and the NRG1 activity on the HER2/HER3 complex can be regulated by a BsAb, limiting the tumor cell expansion. Along this line, some clinical trials are ongoing to define the feasibility of using this therapy in this subset of tumors from different organs, such as NSCLC, PDAC, PC, BC, and others (Table 2).

#### 3.1.3 BsAb Targeting Immune Checkpoint Molecules to Modify the Behavior of the TME

It is well known that the TME plays a key role in downregulating the anti-tumor immune response. This notion is moving the therapeutic target from the tumor cells to the components of TME, such as tumor-associated fibroblasts and regulatory T cells [22,35]. In particular, the antitumor lymphocytes show a behavior of exhausted T cells in the TME due to the plethora of immunosuppressive factors present. To relieve this immunosuppression, the use of therapeutic antibodies to immune checkpoint molecules represents one of the more recent and efficient tools to trigger immune cell response against autologous tumor cells [187,188]. In this context, several mAbs directed to classical immune checkpoints such as CTLA4 or PD1 molecules have been used to treat cancers, leading to unprecedented efficacy in melanomas and NSCLC [187,188,189]. The combo use of anti-CTLA4 and anti-PD1 mAbs suggests that the block of two IC molecules is better than the single targeting of either one [189,190]. To this aim, besides native mAbs, BsAbs have been generated against the CTLA4 and PDL1 or CTLA4 and PD1 and tested in phase II clinical trials (see Table 2) [191,192,193,194,195]. In detail, the KN046 BsAb to CTLA4 and PDL1 molecules block both the CD80/CD86 and PD-1 signaling pathways, ideally improving spatial correlation and therapeutic efficacy [192,196,197,198]. Importantly, the first-in-human study and the phase 1 trial showed that KN046 was safe and effective in patients with advanced solid tumors [193]. It is conceivable that the generation of BsAb to IC molecules may be a more effective and potentially safer treatment option in the management of some solid tumors. This would also reduce the costs of using two distinct mAbs directed to the same molecules. The use of KN046 was assessed in association with chemotherapy in the phase II ClinicalTrials.gov (NCT04054531). It is of note that the rate of patients with relevant treatment-related adverse effects (TRAEs) was 31.0%, similar to what was found in different trials such as CheckMate 227, CheckMate 9LA, and POSEIDON. In addition, the response in non-squamous NSCLC obtained with the BsAb KN046 was comparable with what was observed with mAbs to the IC molecules in combination [192]. The treatment can trigger the production of anti-drug antibodies (ADAs) in 67.9% of treated patients, potentially influencing the efficacy of the BsAb associated with modifications in bioavailability, pharmacokinetic, and pharmacodynamic properties [199]. The rate of this unwanted effect should be further studied to define the factors involved, such as the stability of the BsAb, the presence of impurity, and the influence of the glycosylation.

### 3.2 Chimeric Antigen Receptor Immune Cells: A Powerful Therapeutic Tool against Several Tumors

The chimeric antigen receptor is usually composed of an extracellular domain of a known specificity linked to a transmembrane region through a hinge region derived from a CD8 or IgG4 molecule and an intracellular domain involved in the stimulation and co-stimulation [200,201]. The ectodomain is the portion involved in antigen recognition, as it derives from scFv of an immunoglobulin that targets a cell surface molecule, mainly, if not only, expressed on tumor cells. The endodomain is composed of none, one, or more than one costimulatory domain of costimulatory molecules such as CD27, CD28, ICOS, 4-1BB, and OX40, and a stimulatory domain of CD3, such as the CD3ζ chain or FcγR chain. The effector cells, expressing the CAR, recognize with the extracellular domain scFv, the corresponding antigen expressed on tumor cells. Consequently, the CAR intracellular domain triggers the functional activation of the CAR-bearing cell [200,201]. Intuitively, the triggering through CAR is strong and can lead to the killing of the target cell and the release of pro-inflammatory cytokines, which further increase the anti-tumor effect [200,201]. The transduction of the engineered CAR gene into the desired effector cell is obtained through a retroviral vector or a lentiviral vector or, more recently, via the gene editing tool CRISPR/Cas9 and integration into a specific region of the genome [202,203]. Typically, four generations of CAR have been extensively produced and applied mainly for therapy of some hematological malignancies, such as r/r large B-cell lymphoma (DLBCL), acute lymphoblastic leukemia (ALL), non-Hodgkin lymphoma (NHL), and chronic lymphoblastic leukemia (B-CLL) [203,204], while CAR cells are less efficient in solid tumors [205].

Great limitations in using CAR cells are represented by the expensive nature of the treatment, the time-consuming production, the reduced functionality in a hostile TME, leading to a lack of elimination of tumor cells, and the low persistence in the TME, besides the low concentration within the tumor, at least for solid tumors [205]. One of the main problems with CAR-T cells is represented by the on-target off-tumor toxicity shown in preclinical and clinical studies [206,207,208,209,210]. These effects are due to the lack of unique target antigens on tumor cells not shared with healthy cells. This is less present in the treatment of hematological tumors, in which targeting of CD19 for B-cell lymphoma or B-cell maturation antigen (BCMA) 1 for multiple myeloma (MM) does not lead to such strong damage to healthy B cells or plasma cells [211,212]. One approach to reduce the damage to healthy tissue can be achieved with the generation of CAR-T cells that can distinguish between pathological and healthy cells while recognizing the same EGFR [213]. This has been accomplished by producing CAR-T cells bearing scFv regions of either cetuximab or nimotuzumab. These two anti-EGFR antibodies recognize overlapping epitopes of EGFR but with different affinity for this antigen (cetuximab > nimotuzumab). The feature of nimotuzumab-CAR T is the recognition of the EGFR only when expressed at high levels, such as on glioma cells, but not EGFR at low levels, like on healthy cells [214]. This finding would suggest that it is possible to tune the efficiency of CAR-T cells by building an appropriate chimeric receptor with the ability to recognize some features exclusively shown by tumor cells.

The impairment of functional activities of CAR-T cells in solid tumors is greatly influenced by the suppressive TME [214,215,216,217]. Both tumor and tumor-associated stromal cells, such as mesenchymal stromal cells, can inhibit the TIL effector functions as well as the CAR-cell-mediated cytotoxicity [216,217,218]. Furthermore, the stromal cells can produce a high amount of extracellular matrix protein that impairs the recognition of anti-tumor effectors unless they can degrade this tumor-isolating matrix [219,220,221,222]. To overcome these problems, CAR-T cells expressing heparinase, synthetic (syn) Notch, relaxin-2 (RLN2), an antifibrotic peptide hormone, or treatment with nattokinase can promote intratumor lymphocyte localization and maintain their persistence activation [219,220,223]. To study in detail this latter process, the use of patient-derived organoids is essential. These 3D unconventional culture models can resemble the original tumor and could be tested for the sensitivity to a given CAR-T product, reducing the use of artificial or immunocompromised animal models [224,225,226,227].

### 3.3 CD30 As a Target of CAR Immune Cells against Some Hematological Malignancies

Preclinical and clinical evidence support the usefulness of the CD30-directed CAR-T cells [228]. Two clinical trials, namely NCT02259556 and NCT01316146, using CD30 CAR-T cells in r/r CD30^+^ lymphomas showed low toxicities and some efficacy in a subset of patients [229,230]. In the first clinical trial, 18 patients suffering from r/r HL after a conditioning therapy (fludarabine and cyclophosphamide or gemcitabine, mustargen, cyclophosphamide, or nab-paclitaxel and cyclophosphamide) were treated with autologous CD30-directed CAR-T bearing the costimulatory molecule 4-1BB transduced by lentivirus. It is of note that 5 out of 18 patients showed a history of treatment with BV with progression of the disease. The OR rate was 39% (7 partial responses, PR), and 6 patients had stable disease (SD). The presence of CAR-T cells peaked in the first week after treatment, and the persistence in peripheral blood was detectable for 4–8 weeks. It is of note that the extranodal lesions showed a lower response than those present in lymph nodes, suggesting trafficking of CAR-T cells mainly in lymph nodes and some impairment of this trafficking in the TME of extranodal lesions. Toxicities were not strong, and overall, the treatment was well-tolerated [229].

In the second trial, the administration of CD30-specific CAR-T cells was not preceded by chemotherapy; the CAR-T used as a costimulatory molecule CD28, transduced by γ retrovirus. CAR-T cells have been injected into 9 patients with a history of treatment with chemotherapy (3 or more prior lines) regimens, among whom 7 patients with the antibody BV relapsed after 3 months. The OR rate was 33%, and the treatment was well tolerated. It is of note that none of the patients subjected to infusion of CD30-directed CAR-T cells showed cytokine release syndrome. The presence of CAR-T cells in the periphery peaked within the first week, and detectable levels of these cells were present even after 6 months in 6 out of 9 patients considered [230]. The findings from these two trials would suggest that patients can respond to anti-CD30 CAR-T cells also after using and developing resistance to BV. Evidently, the use of CAR-T cell treatment can be improved by prolonging the persistence and the expansion of effector cells [231,232,233,234,235]. At present, some other clinical trials using CD30 CAR-T cells are ongoing (see Supplementary Table S1).

### 3.4 HER2 As a Target Molecule of CAR Immune Cells to Treat Solid Tumor

The use of HER2 CAR cells to treat several solid tumors, such as glioma, r/r BC, pancreatic cancer, ovarian carcinoma, gastric carcinoma, sarcomas, head and neck squamous cancer, and NSCLC, is under intensive investigation in several ongoing clinical trials (Supplementary Table S2) [236]. It is of note that different effector cells have been transduced with HER2-directed CAR, such as T, NK, and Mo/MΦ [236,237,238,239,240]. These CAR cells can be considered the only therapeutic option for aggressive neoplasia not responding to several lines of conventional therapies. Herein, we describe some examples of these HER2-directed CAR cells. It is of note that T, NK, and monocyte macrophages can be the immune cells bearing the CAR specific for the HER2 transmembrane receptor.

The humanized anti-HER2 or CD47 scFv have been linked to the hinge and transmembrane region of CD8 and the co-signaling molecules 4-1BB and CD3ζ and transduced into the THP1 leukemic cell line as a prototype for monocyte/macrophage. These CAR-M cells showed antigen-specific phagocytosis of ovarian cancer cell line SKOV-3 *in vitro*, stimulating the secretion of several antitumor cytokines by CD8^+^ cytotoxic T lymphocytes (CTL). Also, these CAR-Ms can induce the regression of ovarian tumors, favoring the activation of CD8^+^ T cells [237].

While the elimination of B cells mediated by CAR-T cells is tolerable, as B cells can be substituted by the bone marrow maturation of hematopoietic cell precursors, the elimination of healthy epithelial cells through CAR-T cells recognizing members of the ERBB family can lead to strong toxicity [239,241,242,243]. The engineering of HER2-specific CAR-T with a two-step positive feedback circuit led to the possibility that CD8^+^ CAR-T cells discriminate on the basis of expression of HER2. This circuit was composed of a low-affinity Notch receptor for HER2 that can control the expression of a high-affinity CAR for HER2. The increase of HER2 density led to a cooperative effect that resulted in the discrimination by CAR-T cells between healthy and tumor HER2^+^ cells [242].

## 4 Other Antibody Derivatives or Mimetics of Antibodies to Target Tumor Cells and Trigger the Anti-Tumor Immunity

Several other derivatives of antibodies and other protein-based molecules different from antibodies can be used for anti-cancer therapeutic purposes, including molecular glue-antibody conjugates (MACs), dual payload antibodies, immunocytokines, affibodies [62,244,245,246,247], designed ankyrin repeat proteins (DARPin), anticalins, monobodies, and fynomers [248,249,250,251,252]. Overall, these molecules can interact with the target specifically, like antibodies, but they do not show the molecular structure of an antibody. Some of these molecules can be conjugated with antibodies (MACs) or are chimeric molecules of antibody and cytokine (immunocytokine). Herein, we will focus on some of these new drugs, such as MACs, dual-payload antibodies, and affibodies, while extensive and detailed reviews of others have been published [250,251,252]. Typical examples of these tools have been listed in the Table 3 and some of them will be considered in detail below.

**Table 3:** Representative examples of MACs and other antibody and non-antibody proteins directed against some key molecules for putative antitumor immunotherapy in some hematological or solid tumors.

Molecular Glue Antibody Degrader	Chemical Structure	Antibody Target	Target Molecule	Linker	Main Clinical Indication	Stage of Developing/ Clinical Trial/Phase
Pertuzumab ORM-5029	Degrader SMol006 derived from CC-885	HER2	GSPT1	Peptide linker Val-Cit PABC	HER2^+^ solid cancers	NCT05511844/I
Gemtuzumab onto an IgG1 Fc with N297A variant to inhibit FcγR binding ORM-6151	Degrader SMol006 derived from CC-885	CD33	GSPT1	β-glucuronic acid	r/r AML MDS	NCT06419634/I
LegoChem MACs	Drug 34	EGFR	ND	β-glucoronide	EGFR^+^ cancers	preclinical
Molecular glue antibody stabilizer	Chemical structure	Antibody target	Target molecule	Linker	putative clinical indication	Stage of application
Phosphoantigen-antibody conjugates	Phosphoantigen of XS58	CD20, HER2, CD123, 5T4	BTN3A1 and BTN2A1	Peptide linker Clevable linker	Different types of cancer	preclinical
Other antibody conjugates and targeting	Chemical structure	Antibody target	Target molecule	Linker	putative clinical indication	Stage of application
CD30-BV-ZA	Phosphoramidate bond to brentuximab vedotin Dual payload antibody	CD30	β-tubulin and butyrophilin	No linker	HL CD30^+^ lymphoma cell lines	Preclinical
CET-ZA	Phosphoramidate bond to anti-EGFR	EGFR	butyrophilin	No linker	CRC	preclinical
CET-DC315 Cetuximab Aminothiazole analog of zoledronic acid	Phosphoramidate bond to anti-EGFR	EGFR	butyrophilin	No linker	CRC	preclinical
Affibody ZHER2:342-36FSY-CR8		HER2	Cyclin K		HER2^+^ cancers	preclinical

AML: acute Myeloid Leukemia; BTN3A1, butyrophilin 3A1; BTN2A1, butyrophilin 2; BV: Brentuximab Vedotin; Cet: Cetuximab; DDB1, damaged DNA binding protein 1; GSPT1, G1 to S phase transition 1; MDS: Myelodisplastic Syndrome; ZA: Zoledronic Acid.

### 4.1 Molecular Glue Degrader Antibody Conjugates

MACS can be considered as a class of ADC with molecular glues as payload involved in the killing of target cells, different from typical cytotoxic drugs present in ADC. These payloads can function as degraders or stabilizers of the target molecule and may overcome the resistance induced versus conventional ADC and/or target intracellular proteins otherwise undruggable. Two clinical trials are ongoing using MACS, namely ORM-5029 and ORM-6151 (NCT05511844, phase I and NCT06419634, phase I). The ORM-5029 is composed of an anti-HER2 antibody pertuzumab, the degrader SMoI006, and the valine-citrulline-PABC linker. The glue payload is the SMoI006, derived from the CC-885, which targets the G1 to S phase transition 1 (GSPT1) protein to the E3 ubiquitin ligase, leading to the GSPT1 ubiquitination and degradation via 26S proteasomes. As GSPT1 is essential for the termination of translation of mRNA, ribosome recycling, nonsense-mediated decay, and cell cycle regulation [62], its degradation leads to cell death. It is of note that ORM-5029 suppressed tumor cell growth similarly to T-DXd and better than T-DM1. Also, the combo of ORM-5029 and T-DXd was better than T-DXd alone, suggesting the future possibility of using classical anti-HER2 ADC together with MACs.

The clinical trial NCT06419634 studies the BMS-986497 (ORM-6151) as monotherapy in double and triple combo with azacitidine and venetoclax in r/r patients suffering from acute myeloid leukemia (AML) or myelodysplastic syndrome (MDS). The BMS-986497 is composed of gemtuzumab (anti-CD33) onto an IgG1 Fc with N297A variant to inhibit FcγR binding and the same degrader SMol006 of ORM-5029 targeting the GSPT1. This drug showed strong activity against CD33^+^ AML cell lines (also in Mylotarg-resistant AML193 and Kasumi6 cell lines) and primary AML blasts from patients with a minimal cytotoxicity to hematopoietic progenitor cells (less than Mylotrag), suggesting a good safety profile. In a xenograft model of MV4-11 AML, ORM-6151 administration was better than Mylotarg, the soluble SMoI006 degrader and CC-990009 degrader, indicating that BMS-986497 is a suitable candidate for AML therapy in cases resistant at least to Mylotarg.

Other molecular glue degraders have been recently well reviewed [62,253,254,255]. The main molecular mechanism at the basis of these new therapeutic tools is the specific interaction with essential mediators of cancer cell life that leads to degradation of target proteins and consequent block of cell proliferation. Other molecules, such as CC-92480, CC-220, and CC-99282, target the IKAROS family transcription factors IKZF1/3 involved in the proliferation of tumor B cells in multiple myeloma and lymphomas. These molecules are under investigation in several clinical trials, mainly in r/r MM and NHL [256]. The generation of these drugs started in 2010 with the discovery of cereblon (CRBN), the target of thalidomide [257], and it was accompanied by the selection of proteolysis-targeting chimeras (PROTAC). These molecules target proteins for degradation. The molecular glues are monovalent and small molecules that trigger and stabilize the link between the target protein and the E3 ligase, while PROTACs are bifunctional molecules able to interact on one hand with the E3 ligase and on the other with the target protein. The E3 ligase targets the complex to the polyubiquitination and consequent degradation by the 26S proteasome.

### 4.2 Molecular Glue Stabilizer–Antibody Conjugates: the Phosphoantigen Example

In addition to molecular glue degraders, which determine the inactivation of the target molecule, there are substances that, after the interaction with the target molecule, can improve its function, leading to an increased effect responsible for the inactivation of the target cells or the triggering of an immune response [258,259]. Indeed, some molecular glue stabilizers are phosphoantigen (pAgs) antibody conjugates, such as rituximab (anti-CD20), trastuzumab (anti-HER2), anti-CD123, and anti-5T4 antibodies, via cleavable linkers and CXCR4 targeting moiety with FK506 [254]. Typically, pAg can stabilize the heterodimers between the intracellular domains of the butyrophilin 3A1 (BTN3A1) and butyrophilin 2A1 (BTN2A1) [260,261]. These transmembrane proteins can favor the interaction with the Vδ2^+^ γδT cells, triggering a strong activating signal leading to the production of antitumor cytokines and release of perforins and granzymes. These factors can kill the target cell expressing the BTN3A1 and BTN2A1 complexes [260]. It has been recently shown that pAgs can be conjugated to several tumor-targeting antibodies, including the anti-CD20 rituximab, the anti-HER2 trastuzumab, and anti-CD123 or anti-5T4 antibodies through a cleavable linker [261]. For example, the anti-CD20-pAg (XS58) antibody preincubated with CD20^+^ EBV-related Burkitt lymphoma Raji cells and co-cultured with PBMC for a short period of time (6 h) can trigger the release of IFNγ and the expression of CD107a (lysosomal associated membrane protein, LAMP, 1) on γδT cells. These are two effector molecules typical of anti-tumor lymphocytes. The expression of LAMP1 at the cell surface is an indirect marker of the degranulation and cytolytic process, as LAMP1 is present in intracellular vesicles together with perforins and granzymes, these latter factors are the final pro-cytolytic molecules responsible for the killing of tumor target cells [260].

### 4.3 Molecular Glue Stabilizer–Antibody Conjugates: the Aminobisphosphonate Example

**Figure 5:**
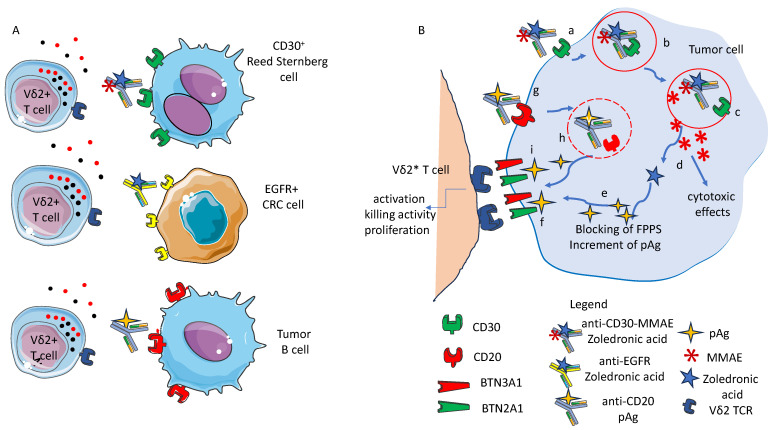
**Triggering of Vδ2^+^ T cells through molecular glue antibody conjugates (MACs) or ADCs conjugated to aminobisphosphonates.** (**A**). Schematic representation of the recognition by Vδ2 T cells of HL cells with BV conjugated to zoledronic acid (ZA) (upper), EGFR^+^ colon rectal carcinoma (CRC) cells with the antibody cetuximab conjugated to ZA (middle), or the anti-CD20 antibody linked to the molecular glue stabilizer phosphor antigen (pAg) (lower). (**B**). Mechanism of action of the anti-CD20-pAg MACs and BV-ZA. The BV-ZA recognizes the CD30 antigen (a), the CD30-BV-ZA complex enters the cell (b), in endolysosomes (c) the drug monomethyl auristatine E (MMAE) and the ZA (d) are released. The cytotoxic drug MMAE triggers the cytotoxic effect, while the ZA inhibiting the FPPS, leads to the accumulation of pAg (e). These pAgs can stabilize the butyrophilins (BTN3A1/BTN2A1) and activates Vδ2 T cells (f). The anti-CD20-pAg mAb binds to CD20 (g), and after the endocytosis (h), the pAg is released, favoring the stabilization of butyrophilins (BTN3A1/BTN2A1), triggering Vδ2 T cells (i). The pAgs leads to proliferation of Vδ2 T cells and trigger tumor cell killing. This figure has been designed from the templates of Anatomy and Human Body and Cellular Biology series of Smart Servier Medical Art (https://smart.servier.com/).

The molecular glue stabilizer represented by pAg stimulates the triggering of Vδ2^+^ γδT cells because it has been shown that pAg, such as the isopentenylpyrophosphate (IPP) or its isomers dimethylallyl pyrophoshates (DMAPP), can interact with the intracellular domains of the above mentioned BTN3A1 and BTN2A1 and presenting the pAg to the Vδ2 γδT cells [261,262] (Fig. 5). The pAgs are generated inside the target cell through the mevalonate pathway, responsible for the synthesis of cholesterol [262,263,264]. It is of note that an increment of pAgs in a specific cell can be achieved by the treatment with amino-bisphosphonates (N-BPs) like zoledronic acid [265,266,267,268,269]. This compound can impair the functional activity of the farnesyl-pyrophosphate synthase (FPPS) [270,271], limiting the use of the IPP and DMAPP. This blocking leads to accumulation of pAgs inside the target cell [263,264]. If the target cell expresses the appropriate butyrophilin, the presentation of pAgs leads to Vδ2^+^ γδT cell triggering. This property of N-BPs is well known, and it has been utilized to stimulate anti-tumor immune response against colorectal carcinomas (CRC) organoids using the anti-EGFR antibody cetuximab covalently linked with zoledronic acid, termed Cet-ZA [272]. *In vitro* experiments showed that this ADC can target tumor-associated fibroblasts [273], leading to the elimination of this immunosuppressive cell population. Also, it has been shown previously that different N-BPs can be linked to the cetuximab, such as risedronic acid or ibandronic acid, namely Cet-RIS and Cet-IBA [274]. These conjugates showed *in vitro* similar functional features of Cet-ZA, and they should be tested *in vivo* to define the tolerability and feasibility of their possible administration in a clinical setting. More recently, an aminothiazole analog of zoledronic acid termed DC315 has been selected and conjugated to cetuximab [275] to improve the drug-antibody ratio of the N-BP linked to the anti-EGFR. This new conjugate, termed CET-DC315, showed a very high DAR (DAR = 23) compared to the previously described N-BPs conjugates (DAR = 2–4) and displayed a good antitumor effect similar to that of the other conjugates. It is still to be defined in *in vivo* models whether this new ADC can show a better anti-tumor effect due to the higher quantity of N-BPs that can be targeted to tumor cells. Overall, these findings suggest that either using directly pAgs-linked or indirectly N-BPs-linked antibodies, it is possible to stimulate the immune response of a specific subset of anti-tumor lymphocytes that can be localized into epithelial layers [276,277].

### 4.4 Other Molecular Glue Stabilizer–Antibody Conjugates

Another molecular glue-stabilizer is the anti-CD184-FK506 complex developed by Ambrrx Inc. (https://www.adcreview.com/drugmap/cd184-fk506/). In this case, the clinical application is not in anti-tumor targeting but in improving the immunosuppressive effects of the FK506 drug, also named as tacrolimus. This drug is able to bind to the FK506 binding protein 12 (FKBP12) forming a complex stably interacting with calcineurin blocking its activity. This would suggest that molecular glues can be used in the clinic for different purposes antineoplastic and immunosuppressive in autoimmune diseases [278,279].

### 4.5 Dual Payloads ADC Targeting of Multiple Intracellular Targets

The classical ADC use the specifity of the mAb to deliver a cytotoxic drug to the tumor cells expressing the recognized antigen. Of course, the tumor cells targeted can be selected to become adapted to the drug carried by the ADC. It is reasonable that two drugs carried simultaneously to the same target cell can exert a stronger toxic effect than a single drug as it happens using combo of drugs to treat tumors. Indeed, ADCs have shown good clinical efficacy but with an incomplete response, generation of resistance, and some adverse effects [110,280]. Thus, several studies have been performed to allow the conjugation with more than one payload of mAbs to target different essential molecules for cancer cell growth [281,282,283,284,285]. The dual-payload ADCs can increase the efficacy of antibodies with a single payload and promote a synergistic effect, reducing the generation of resistant cells. Indeed, the effect on two molecules with distinct and independent activities can lead to cancer cell death through different pathways [286,287,288]. In this context, it has been shown that it was possible to achieve precise delivery through a detailed optimization of the linker design of an antibody conjugated with exatecan mesylate (EXA) and triptolide (TPL) drugs. The former is a topoisomerase I inhibitor with a higher cell permeability of deruxtecan, while the latter can interfere with HSP70, leading to a strong inhibition of RNA polymerase II [289,290]. The dual-payload TROP2-targeting ADC, hRS7-EXA+TPL (KH815), can react well with TROP2^+^ cells similarly to the native anti-TROP2 antibody. Furthermore, the dual-payload ADC can exert a more persistent antitumor response in patient-derived xenografts (PDX), and it can overcome the resistance to other anti-TROP2 ADCs linked to deruxtecan [288]. Also, trastuzumab was conjugated with two anti-microtubule drugs, such as VcMMAE-SMCCDM1 generating a dual-payload antibody for an optimal antitumor effect. These two drugs were linked to the anti-HER2 antibody with two different linkers: valine-citrulline (VC) and succinimidyl-4-(N-maleimidomethyl) cyclohexane-1-carboxylate (SMCC). Importantly, this new anti-HER2 ADC can show a stronger antitumor effect as well as a better penetration and retention of this target-specific therapeutic agent compared with other anti-HER2 antibodies [287]. Finally, an anti-CD276 antibody conjugated with an immunoregulating Toll-like receptor 7/8 agonist (Imiquimod, IMQ) and the microtubule inhibitor MMAF could be used to target triple-negative BC cells [286]. The CD276 is also known as B7-H3, and it is an inhibitory ligand present on different tumor cells that can deliver an inhibiting signal into anti-tumor effector lymphocytes [291]. It is of note that the dual-payload anti-CD276 antibody can exert a strong antitumor cytotoxic effect *in vitro* on triple-negative BC cells. Further, the antitumor response in an *in vivo* model using triple-negative BC cells was more pronounced with the dual-payload antibody compared to the anti-CD76 antibody linked to just another payload. This effect was due to a more evident infiltration of tumors with effector lymphocytes such as CD8^+^ and NK cells, together with a strong phagocytosis of tumor cells. These findings suggest that the dual payload antibody can recruit immune effector cells to the tumor site together with the delivery of a strong cytotoxic anti-tumor effect. The generation of dual payload antibodies is not easy, and several matters in generating the optimal chemical conditions to obtain these potent drugs should be faced [282,283]. Another approach to the generation of an antibody linked to two drugs can be considered the conjugation of the anti-CD30 BV to the N-PB ZA recently reported generating the Bre-Ved-ZA (BV-ZA) antibody [285]. This new ADC can associate the anti-lymphoma effect due to the BV with the possibility of stimulating the expansion and the activation of cytolysis of Vδ2^+^ T cells against CD30^+^ tumor cell lines. Indeed, the BV-ZA ADC can exert a more potent direct cytotoxic effect than the BV. Also, the processing of the ZA inside the CD30^+^ cell lines can lead to stimulation of Vδ2^+^ T cells and consequent increment of the antitumor effect [285].

### 4.6 Affibodies As Mimetic of Antibodies for Specific Recognition of Key Molecular Components

The target specificity shown by mAbs is the molecular feature at the basis of the rationale to treat tumors with ADC, as shown above. However, mAbs are quite large glycosylated proteins (about 150,000 daltons) that show high costs and production complexity, poor tissue penetration, and stability, besides immunogenicity and adverse reactions. A possible alternative to mAbs mimicking their antigen specificity is the engineered proteins as affibodies, characterized by a low molecular weight of 6500–7000 daltons. Indeed, affibody molecules are based on a robust molecular scaffold with a three-helix domain and characterized by a strong solubility and resistance to proteolysis by themselves or after chimeric fusion with other proteins [292,293]. Affibodies can be produced specifically against surface receptors expressed by tumor cells to target these cells but also to develop imaging tracers that allow the non-invasive assessment of the localization of tumor cells throughout the body [294,295,296,297,298]. The chemical structure of an affibody (ABY) specific for the B7-H3 surface molecule has been improved and optimized (generating the RESCA-B7H3-BCH) to make some clinical translational and basic studies. The ABY named [^68^Ga]Ga-B7H3-BCH was specifically up taken by B7-H3 transfected cell lines, as this uptake was inhibited by the use of unlabeled precursors. The PET imaging indicated that the affibody was specifically localized to multiple xenograft models, and in a cohort of 20 patients with malignant tumors, the [^68^Ga]Ga-B7H3-BCH can be well taken up by primary and metastatic lesions. The identification of tumor lesions was better than that of fluorine-18 fluorodeoxyglucose (18F-FDG) in overall diagnostic efficacy for tumors. These findings would suggest that this modified ABY may improve the overall clinical staging by analyzing the therapeutic response and the insurgence of resistant lesions [288]. Also, HER2 and HER3 molecules can be a good target for affibodies used for imaging [292,293,294,295,296]. Indeed, the identification of HER2^+^ BC is based on the data from IHC and/or *in situ* fluorescence hybridization (FISH). The expression of HER2 is heterogeneous, and the clinical results upon targeting of this molecule are dependent on the correct identification of HER2^+^ patients [113,114,297,298]. The inter- and intratumoral heterogeneity of HER2 expression analysis is a key step to maximize the patient benefit. Affibodies have been extensively used in imaging studies [299], and a lot of patients have been studied with these new tools for imaging. In particular, the ABY-025 (tezatabep matraxetan), an affibody molecule of 7.55 kDa, binds to human HER2 specifically and at picomolar affinity [300]. ABY-025 recognizes a portion of the HER2 receptor different from those identified by some of the therapeutic anti-HER2 antibodies, such as trastuzumab and pertuzumab. The administration of this ABY did not lead to side effects or to the generation of anti-ABY antibodies for the period of 6 weeks, and it was efficient in identifying HER2^+^ lesions [299,300,301]. Overall, the ^111^In-ABY-025 was safe for the PET and SPECT diagnostic analyses independently of the ongoing anti-HER2 treatment. Finally, the affibodies have been used to generate affibody-drug conjugates against the HER2 or HER3 molecules (see Table 3) with the aim of blocking the HER2/3-mediated signaling [253,302].

In this context, the rationale is to use small molecules such as ABY as flexible molecules to link payloads targeting key biological molecules of a tumor target cell. The affibody named Z_HER2:342_-36_FSY-Cys_ (FSY, fluorosulfate-L-tyrosine) was linked to the cyclin K degrader CR8 and a disulfide-containing cleavable linker [253], generating the conjugate Z_HER2:342_-36_FSY_-CR8. This can self-assemble by a covalent bond into the affibody-drug conjugate nanoagent (ADCN). This compound can target HER2 on ovarian cancer cells, increasing the concentration of the drug CR8 inside the tumor cell, leading to a strong antitumor effect better than that of non-covalent linked affibody [253]. It is of note that the CR8 is a cyclin-dependent kinase (CDK) inhibitor, and its concentration inside a cell is limited without the antibody carrier [303]. Similarly, the targeting of HER3 with polyvalent ABY on a panel of cell lines improved the inhibition of NRG-induced HER3 and Akt phosphorylation, leading to inhibition of cancer cell growth [302]. In addition, HER3 affibodies reduced the progression of ovarian xenograft tumor models as a single agent or in combination with carboplatin [304].

## 5 Challenges and Possible Solutions for an Optimal Targeting of Cancer Cells with mAbs and Derivatives

The knowledge of the complexity of cell-to-cell cross-talk, together with the strong heterogeneity of tumor cells obtained by applying the OMICS techniques even at the single-cell level, gives an intricate scenario to choose the right therapeutic tool. Also, ADCs, BsAb, and CAR cells show a strong clinical efficacy if the target molecule is well expressed on tumor cells. The selection of resistant tumor cells, which are selected within the TME starting from cancer stem cells’ phenotypic features that are shaped during their differentiation, leads to the need to identify novel targets and generate the corresponding ADC, BsAb, or CAR immune cell. As briefly mentioned before, the generation of optimal ADCs, BsAbs, and CAR cells to a specific target takes a long time of preclinical and clinical studies. The huge amount of information coming from the genomics and proteomics analyses of different cohorts of patients all over the world represents the attempt to pull together patients in specific groups to plan a treatment that is valid not only for the single specific patient. But it is evident that some treatments are not efficient in all the patients suffering from the same cancer disease, as the features of cancer cells can be similar in different patients, but the response of the single patient is related to both the features of the tumor and also the host immune response, besides the comorbidities of that particular patient [100,149,171,188,228].

To speed up the analysis and the approval of novel drugs, programs from the FDA and EMA are accelerating the development and review of drugs designed to treat cancer and that may fill an unmet medical need. This process is based on an enhanced interaction between the proponent of the drug that covers an unmet need and the FDA/EMA throughout the development of the drug, the possibility of making smaller and fewer trials to support the approval, and the manufacturer being able to submit completed sections of the study on a drug before finishing the complete analysis. On the other hand, the regulatory body should give a reply in a short time.

Also, the production of therapeutic mAbs as well as the use of biosimilars is a key point in the clinical application [305,306,307]. Usually, biosimilar are produced by a different clone and/or processes with different rate of glycosylation with respect to the the original therapeutic mAb although the formulations are more homogenous for pH and excipient preferences regardless of formulation concentration, drug product presentation, and route of administration [308,309], Also, the cost of production using batch processing which remains the backbone of the mAbs manufacturing can be markedly reduced with continuous processing saving up to 35% of the costs [310]. To improve the production, machine learning approaches for protein expression optimization have shown particular promise [311,312] together with process analytical technology using inline, online, or at-line sensors [313].

To further increase the effectiveness of mAbs, some recent ADC engineering advances, ultra-high-DAR linker-polymer hybrids, and *in-situ* self-assembled mAb–nanodrug depots [314,315,316,317,318]. Some mAbs target PDL1, inducing entry into the cell, leading to a reduction of PDL1 expression. This event is followed by the recycling of the PDL1 at the cell surface (see Fig. 2 for details). This re-expression can favor the immunosuppression due to PDL1-PD1 interactions with a lesser activation and thereby fewer therapeutic effects [319]. It has been proposed to link the RGD peptides and PDL1 antibodies with the poly (L-glutamic acid) nanoparticles (NPs) to generate a nanochimera targeted to the integrin αvβ3, a molecular target of the RGD sequence [316]. After optimization of the ratio of RGD peptide and anti-PDL1 antibody to generate a quadrivalent PDL1-RGD complex that can inhibit 2.5 times better the recycling of the PDL1 molecule, triggering localization to lysosomes. The nanochimera formed a terpolymer complex with αvβ3 integrin and PDL1 molecules at the cell surface of cancer cells, and the PDL1 was markedly degraded inside lysosomes, thereby enhancing the durability of the immune response against tumor cells. These anti-PDL1-RGD-NPs can increase the production of anti-tumor cytokines such as IFNγ and TNFα with an increase of granzyme B expression and consequent antitumor immune response in a murine *in vivo* model. This approach underscores this novel strategy to improve the therapeutic effectiveness of anti-PDL1 mAbs. Regarding the advancement in the production of ADC due to several new technologies and methods, click chemistry is an example of stereospecific reactions producing minimal byproducts under mild conditions. These are ideal for creating complex molecules such as drugs and bioconjugates [314,320,321,322]. Indeed, an antibody targeting an intracellular molecule has been encapsulated inside a nanogel, while a second antibody targeted an extracellular antigen. This system was programmed to release the cargo antibodies to specific cells, engaging intracellular targets [321]. In particular, three functional antibodies, such as anti-nuclear pore complex targeting the nucleus, anti-TOM20 targeting mitochondria, or anti-phospho AKT antibodies targeting cytoplasm, have been encapsulated in nanocarriers bearing anti-HER2 antibody Trastuzumab. These complexes penetrated into HER2^+^ BC cancer cells, inhibiting the corresponding cellular component recognized by the antibody delivered. This system enables precise tracking of desired subcellular targets with a two-step verification process, presenting potential for reliable antibody-based delivery of biologics.

## 6 Conclusions

With the discovery of the method to generate mAbs, it became clear soon that the potential applications of these protein-based tools in research and afterward in a clinical setting should be almost unlimited. It is evident that the use of mAbs and their derivatives in basic, translational, and clinical research has allowed unbelievable successes and progress. Besides these unprecedented results in cancer therapy, it is clear that the generation of resistant tumor cell clones can impair the therapeutic efficacy of mAbs. The use of antibodies as a carrier of cytotoxic drugs has improved the clinical results, but again, the heterogeneity of the tumor and its immunosuppressive TME favor the overgrowth of resistant tumor cells, leading to tumor relapse. The possibility of carrying molecular glues through antibodies has raised the possibility of targeting intracellular key target molecules, such as transcription factors, and increasing the therapeutic effect of otherwise undruggable compounds. The engineering of CAR immune cells with the specificity of a mAb has allowed the treatment of relapsed/refractory patients, but the long-lasting function of these potent antitumor effector cells is again impaired by the possibility of generating tumor-resistant cells. Again, the TME plays a central role in blocking the antitumor effect of CAR cells. The future perspectives of the use of antibodies and their derivatives reside, on one hand, on the further knowledge of the TME using omics approaches and, on the other, on the selection of antibody-based tools with more than one drug linked or with a cytotoxic drug and another molecule able to stimulate the immune system response and/or impair or reprogram the immunosuppressive features of the TME. The possibility of targeting several biochemical pathways at the same time will reduce the emergence of resistant cells, and it will allow us to target tumor cells. The appropriate treatment schedules with combos of antibodies and/or their derivatives together with chemotherapeutics will increase the therapeutic effects of each other and reduce the possibility of overgrowth of insensitive tumor cells. In this context, the use of antibody tools against not only classical ICI such as CTLA4 and PD1/PDL1 but also inhibitory receptors of both innate and adaptive immune cells could improve the anti-tumor immunity [323,324,325].

## Data Availability

Not applicable.

## Supplementary Materials

The supplementary material is available online at https://www.techscience.com/doi/10.32604/or.2026.078483/s1.

## Abbreviations

ABY	affibody
ADAM10	a disintegrin and metalloproteinase domain 10
ADC	antibody drug conjugate
ADCC	antibody dependent cellular cytotoxicity
ADCN	affibody-drug conjugate nanoagent
ALCL	anaplastic large cell lymphoma
ALL	acute lymphoblastic leukemia
AML	acute myeloid leukemia
ATL	adult T-cell leukemia/lymphoma
BC	breast carcinoma/cancer
BCMA	B-cell maturation antigen
BsAb	bispecific antibody
BTN3A1	butyrophilin 3A1
BTN2A1	butyrophilin 2A1
BV	brentuximab vedotin
CAR	chimeric antigen receptor
CD	cluster of differentiation
CDC	complement dependent cytotoxicity
CDK	cyclin dependent kinase
Cet	cetuximab
CLL	Chronic lymphocytic leukemia
CR	complete response
CRBN	cereblon
CRISPR	Clustered Regularly Interspaced Short Palindromic Repeats
CRC	colorectal carcinoma
CRS	cytokine release syndrome
CTL	cytotoxic T lymphocytes
CTLA4	Cytotoxic T-Lymphocyte Antigen 4
CXCR4	C-X-C motif chemokine receptor 4
Cu	cuprum
DAR	drug antibody ratio
DARPins	designed ankyrin repeat proteins
DFS	disease free survival
DLBCL	diffuse large B-cell lymphoma
DM1	mertansine/emtansine
DMAPP	dimethylallyl pyrophoshates
DNA	deoxyribonucleic acid
DOTA	1,4,7,10-tetraazacyclododecane-1,4,7,10-tetraacetic acid
EBV	epstein barr virus
EGFR	epidermal growth factor receptor
EMA	European Medicines Agency
ERBB	Epidermal Growth Factor Receptor family
EXA	exatecan mesylate
Fab	fragment antigen binding
FASL	FAS ligand
FDA	food and drug administration
FISH	fluorescence situ hybridization
FKBP12	FK506 binding protein 12
GSPT1	G1 to S phase transition 1
HER2	human epidermal growth factor receptor 2
HER3	human epidermal growth factor receptor 3
HL	Hodgkin lymphoma
HTLV-1	human T-cell leukemia virus type 1
IBA	ibandronic acid
ICAM1	Intercellular adhesion molecule 1
ICI	immune checkpoint inhibitor
Ig	immunoglobulin
IFN	interferon
IHC	immunohistochemistry
IMQ	imiquimod
IPP	isopentenylpyrophosphate
LAMP1	lysosomal associated membrane protein 1
LFA1	lymphocyte function associated antigen 1
LFA3	lymphocyte function associated antigen 3
Lu	lutetium
mAbs	monoclonal antibodies
MACs	molecular glue antibody conjugates
mBC	metastatic breast carcinoma
MDS	myelodysplastic syndrome
MF	mycosis fungoide
MAPK	mitogen-activated protein kinase
MMAE	monomethyl auristatin E
MMAF	monomethyl auristatin F
Mo/MΦ	monocyte/macrophage
N-BP	aminobisphosphonate
NHL	non-Hodgkin lymphoma
NRG	neuregulin
NFkB	nuclear factor kappa B
NK	natural killer
NOTA	1,4,7-triazacyclononane-1,4,7-triacetic acid
NSCLC	non-small cell lung cancer
NSG	NOD SCID gamma
ORR	overall response rate
pAg	phosphoantigen
PBMC	peripheral blood mononuclear cells
PC	prostate carcinoma
PDAC	pancreatic adenocarcinoma
PD1	programmed cell death protein 1
PDL1	programmed cell death protein ligand 1
PDO	patient-derived organoid
PDX	patient-derived xenografts
PET	positron emission tomography
PI3K	phospho inositol 3 kinase
PFS	progression-free survival
PR	partial response
PTCL	peripheral T cell lymphoma
RAB11A	Ras-related protein Rab-11A
RIS	risedronic acid
RIT	radio immunotherapy
RLN2	relaxin 2
r/r	relapse/refractory
scFv	single-chain variable fragment
SCID	severe combined immunodeficiency
SCLC	small cell lung cancer
SD	stable disease
SMMC	succinimidyl-4-(N-maleimidomethyl cyclohexane-1carboxylate
SNX10	sorting nexin 10
SPECT	Single Photon Emission Computed Tomography
Tb	terbio
TDXd	trastuzumab deruxtecan
TDM1	trastuzumab mertansine/emtansine
TIL	tumor infiltrating lymphocyte
TNF	tumor necrosis factor
TNFRSF	tumor necrosis factor receptor superfamily
TME	tumor microenvironment
TPL	triptolide
TR	trastuzumab resistant
VC	valine-citrulline
ZA	zoledronic acid
