# Supplementary material for: Monoclonal Antibodies and Derivatives: Therapeutic Tools for Cancer
Source: Oncol Res. 2026 Jun 16;34(7):6. doi: 10.32604/or.2026.078483 (PMC13292028; doi:10.32604/or.2026.078483)
Supplement: Supplementary file 1 [file OncolRes-34-78483-s001.zip › TSP_OR_78483-s001.docx]

Supplemental Table S1. Clinical trials using CD30-directed CAR cells in hematological malignancies

| Study title | NCT number | status | Pathological condition | interventions | sponsor |
| --- | --- | --- | --- | --- | --- |
| [Phase 1 Autologous CD30.CAR-T in r/r CD30+](https://clinicaltrials.gov/study/NCT04526834?intr=CD30%20CAR&page=1&limit=50&rank=1) NHL | NCT04526834 | Active, not recruiting | ALCL, PTCL, ENK/TCL, 2 more | CD30.CAR-T | Tessa Therapeutics |
| [Anti-CD30 CAR-T Therapy in Patients With Refractory/Relapsed Lymphocyte Malignancies](https://clinicaltrials.gov/study/NCT04008394?intr=CD30%20CAR&page=1&limit=50&rank=2) | NCT04008394 | Unknown status | Adult T-Cell Lymphoma/Leukemia, ALCL, Angioimmunoblastic T-cell Lymphoma, 3 more | Anti-CD30 CAR T cells | Union Hospital, Tongji Medical College, Huazhong University of Science and Technology |
| [Allogeneic CD30.CAR-EBVSTs in Patients With Relapsed or Refractory CD30-Positive Lymphomas](https://clinicaltrials.gov/study/NCT04288726?intr=CD30%20CAR&page=1&limit=50&rank=3) | NCT04288726 | Recruiting | ENK/TCL, Nasal Type, cHL | CD30.CAR-EBVST cells | Baylor College of Medicine |
| [Allogeneic CD30 Chimeric Antigen Receptor Epstein-Barr Virus-Specific T Lymphocytes in Relapsed or Refractory CD30-Positive Lymphomas](https://clinicaltrials.gov/study/NCT04952584?intr=CD30%20CAR&page=1&limit=50&rank=4) | NCT04952584 | Withdrawn | ENK/TCL, Nasal Type, cHL | CD30.CAR-EBVST cells | Baylor College of Medicine |
| [Camrelizumab Combined With CD30 CAR-T in the Treatment of Relapsed/Refractory CD30+ Lymphoma](https://clinicaltrials.gov/study/NCT05320081?intr=CD30%20CAR&page=1&limit=50&rank=5) | NCT05320081 | Unknown status | Lymphoma, Relapse/Recurrence | CD30 CAR-T, Camrelizumab | Huazhong University of Science and Technology |
| [CAR T Cells Targeting CD30 Positive Lymphomas (4SCAR30273)](https://clinicaltrials.gov/study/NCT02274584?intr=CD30%20CAR&page=1&limit=50&rank=6) | NCT02274584 | Unknown status | Lymphomas | Anti-CD30 CAR T cells | Peking University |
| [CD30 CAR-T in the Treatment of CD30 Positive Relapsed/Refractory Lymphoma](https://clinicaltrials.gov/study/NCT06850285?intr=CD30%20CAR&page=1&limit=50&rank=7) | NCT06850285 | Recruiting | Lymphoma, B-Cell | CAR gene modified T cells | Shanxi Bethune Hospital |
| [CD30 CAR T-cells Post AutoHSCT for Poor-risk Hodgkin Lymphoma](https://clinicaltrials.gov/study/NCT06617286?intr=CD30%20CAR&page=1&limit=50&rank=8) | NCT06617286 | Not yet recruiting | cHL | CD30 CAR T-cell | New York Medical College |
| [CD30 CAR-T in the Treatment of CD30 Positive Lymphoma](https://clinicaltrials.gov/study/NCT07048353?intr=CD30%20CAR&page=1&limit=50&rank=9) | NCT07048353 | Not yet recruiting | Lymphoma, B Cell Lymphoma, CD30+ Peripheral T-cell Lymphoma | Chimeric antigen receptor gene modified T cells | Shandong Qilu Cell Therapy Engineering Technology Co., Ltd |
| [Safety and Efficacy of Anti-CD20/CD30 CAR-T Cells in Subjects with Relapsed/Refractory Lymphoma](https://clinicaltrials.gov/study/NCT06532643?intr=CD30%20CAR&page=1&limit=50&rank=10) | NCT06532643 | Recruiting | Relapsed/Refractory Lymphoma | anti-CD20/CD30 CAR-T cells, Fludarabine, Cyclophosphamide | Shanghai First Song Biotechnology Co., LTD |
| [An Exploratory Clinical Study Evaluating the Safety and Efficacy of Anti CD30 CAR T Cells in Patients With CD30+ Relapsed/Refractory Lymphoma](https://clinicaltrials.gov/study/NCT05208853?intr=CD30%20CAR&page=1&limit=50&rank=11) | NCT05208853 | Unknown status | HL, NK/T Cell Lymphoma, PTCL, Unspecified, 5 more | Anti-CD30 CAR-T Cell Injection | Zhejiang University |
| [An Exploratory Clinical Study Evaluating the Safety and Efficacy of Intravenous Anti-CD20/CD30-CAR-T Cell Infusion in Relapsed/Refractory Lymphoma Patients.](https://clinicaltrials.gov/study/NCT06519344?intr=CD30%20CAR&page=1&limit=50&rank=12) | NCT06519344 | Not yet recruiting | B Cell Malignancies | anti-CD20/CD30-CAR-T Cells | Shanghai Tongji Hospital, Tongji University School of Medicine |
| [Constitutive IL7R (C7R) Modified Banked Allogeneic CD30.CAR EBVSTS for CD30-Positive Lymphomas](https://clinicaltrials.gov/study/NCT06176690?intr=CD30%20CAR&page=1&limit=50&rank=13) | NCT06176690 | Recruiting | CD30+ DLBCL, ALCL, T Cell and Null Cell Type, ALCL ALK+, 4 more | C7R.CD30.CAR-EBVST cells | Baylor College of Medicine |
| [A Study of CAR T-Cells in Relapsed/Refractory Hematologic Malignancy](https://clinicaltrials.gov/study/NCT06756321?intr=CD30%20CAR&page=1&limit=50&rank=14) | NCT06756321 | Recruiting | Relapsed/Refractory Lymphoma, Relapsed/Refractory Leukemia | anti-CD19-CAR T-cells, or anti-CD30-CAR T-cells, or anti-CD20/CD30-CAR T-cells, Fludarabine, Cyclophosphamide | Affiliated Hospital of Nantong University |
| [A Clinical Research of CD30-Targeted CAR-T in Lymphocyte Malignancies](https://clinicaltrials.gov/study/NCT02958410?intr=CD30%20CAR&page=1&limit=50&rank=15) | NCT02958410 | Unknown status | Leukemia, Lymphoma | Anti-CD30-CAR-transduced T cells | Southwest Hospital, China |
| [Phase 2 Study Evaluating Autologous CD30.CAR-T Cells in Patients With Relapsed/Refractory Hodgkin Lymphoma (CHARIOT)](https://clinicaltrials.gov/study/NCT04268706?intr=CD30%20CAR&page=1&limit=50&rank=16) | NCT04268706 | Active, not recruiting | HL, Adult, HD Recurrent, HD Refractory, 1 more | CD30.CAR-T, Fludarabine, Bendamustine | Tessa Therapeutics |
| [CD30 CAR T Cells, Relapsed CD30 Expressing Lymphoma (RELY-30)](https://clinicaltrials.gov/study/NCT02917083?intr=CD30%20CAR&page=1&limit=50&rank=17) | NCT02917083 | Recruiting | HL, NHL | CAR T Cells | Baylor College of Medicine |
| [CD30 Targeted CAR-T in Treating CD30-Expressing Lymphomas](https://clinicaltrials.gov/study/NCT03383965?intr=CD30%20CAR&page=1&limit=50&rank=18) | NCT03383965 | Recruiting | HL, ALCL | ICAR30 T cells | Immune Cell, Inc. |
| [CAR T-cells Against CD30 (HSP-CAR30) for Relapsed/ Refractory Hodgkin and T-cell Lymphoma.](https://clinicaltrials.gov/study/NCT04653649?intr=CD30%20CAR&page=1&limit=50&rank=19) | NCT04653649 | Unknown status | HL, Adult T Cell Lymphoma | HSP-CAR30 | Fundació Institut de Recerca de l'Hospital de la Santa Creu i Sant Pau |
| [Autologous CD30.CAR-T in Combination With Nivolumab in cHL Patients After Failure of Frontline Therapy](https://clinicaltrials.gov/study/NCT05352828?intr=CD30%20CAR&page=1&limit=50&rank=20) | NCT05352828 | Active, not recruiting | cHL, HD Refractory, HD Recurrent | Nivolumab, Autologous CD30.CAR-T Fludarabine, 1 more | Tessa Therapeutics |
| [GSL Synthetase Inhibitor Eliglustat Combined With CD30 Target Immunotherapy for the Treatment of  CD30+ Lymphoma](https://clinicaltrials.gov/study/NCT07138547?intr=CD30%20CAR&page=1&limit=50&rank=21) | NCT07138547 | Not yet recruiting | Lymphoma | Eliglustat, CD30 target immunotherapy | Chinese PLA General Hospital |
| [Study of PD-1 Inhibitors After CD30.CAR T Cell Therapy in Relapsed/Refractory Hodgkin Lymphoma](https://clinicaltrials.gov/study/NCT04134325?intr=CD30%20CAR&page=1&limit=50&rank=22) | NCT04134325 | Active, not recruiting | Relapsed HL, Refractory HL | Nivolumab, Pembrolizumab | UNC Lineberger Comprehensive Cancer Center |
| [CD30 CAR for Relapsed/Refractory CD30+ T Cell Lymphoma](https://clinicaltrials.gov/study/NCT04083495?intr=CD30%20CAR&page=1&limit=50&rank=23) | NCT04083495 | Recruiting | PTCL | ATLCAR.CD30 T cells, Bendamustine, Fludarabine, 1 more | UNC Lineberger Comprehensive Cancer Center |
| [CAR-T for R/R B-NHL](https://clinicaltrials.gov/study/NCT03196830?intr=CD30%20CAR&page=1&limit=50&rank=24) | NCT03196830 | Unknown status | Relapsed NHL, Refractory NHL, CAR -T CD19/CD20/CD22/CD30 | CAR-T | The First Affiliated Hospital of Soochow University |
| [CD30 CAR for CD30+ NSGCT](https://clinicaltrials.gov/study/NCT05634785?intr=CD30%20CAR&page=1&limit=50&rank=25) | NCT05634785 | Active, not recruiting | Germ Cell Tumor, Non-seminomatous Germ Cell Tumor | ATLCAR.CD30 Cells, Cyclophosphamide, Fludarabine | UNC Lineberger Comprehensive Cancer Center |
| [Combination CAR-T Cell Therapy Targeting Hematological Malignancies](https://clinicaltrials.gov/study/NCT03125577?intr=CD30%20CAR&page=1&limit=50&rank=26) | NCT03125577 | Recruiting | B-cell Malignancies | 4SCAR19 and 4SCAR22, 4SCAR19 and 4SCAR38, 4SCAR19 and 4SCAR20, 3 more | Shenzhen Geno-Immune Medical Institute |
| [T Cells Expressing a Fully-Human Anti-CD30 Chimeric Antigen Receptor for Treating CD30-Expressing Lymphomas](https://clinicaltrials.gov/study/NCT03049449?intr=CD30%20CAR&page=1&limit=50&rank=27) | NCT03049449 | Completed  [WITH RESULTS](https://clinicaltrials.gov/study/NCT03049449?intr=CD30%20CAR&page=1&limit=50&tab=results&rank=27) | Lymphoma, LCA, Enteropathy-Associated T-Cell Lymphoma, Lymphoma, DLCL, 2 more | Anti-Tumor Necrosis Factor (TNF) Receptor Superfamily Member 8 (CD30) CAR T cells, Cyclophosphamide, Fludarabine | National Cancer Institute (NCI) |
| [CD30-directed Chimeric Antigen Receptor T (CART30) Therapy in Relapsed and Refractory CD30 Positive Lymphomas](https://clinicaltrials.gov/study/NCT02259556?intr=CD30%20CAR&page=1&limit=50&rank=28) | NCT02259556 | Recruiting | HL, NHL | CART30 | Chinese PLA General Hospital |
| [Study of CD30 CAR for Relapsed/Refractory CD30+ HL and CD30+ NHL](https://clinicaltrials.gov/study/NCT02690545?intr=CD30%20CAR&page=1&limit=50&rank=29) | NCT02690545 | Active, not recruiting | Lymphoma, NHL, Immune System Diseases, 5 more | ATLCAR.CD30 cells | UNC Lineberger Comprehensive Cancer Center |
| [Administration of T Lymphocytes for Prevention of Relapse of Lymphomas](https://clinicaltrials.gov/study/NCT02663297?intr=CD30%20CAR&page=1&limit=50&rank=30) | NCT02663297 | Active, not recruiting  [WITH RESULTS](https://clinicaltrials.gov/study/NCT02663297?intr=CD30%20CAR&page=1&limit=50&tab=results&rank=30) | HL, NHL, 6 more | ATLCAR.CD30 cells | UNC Lineberger Comprehensive Cancer Center |
| [EBV CTLs Expressing CD30 Chimeric Receptors For CD 30+ Lymphoma](https://clinicaltrials.gov/study/NCT01192464?intr=CD30%20CAR&page=1&limit=50&rank=31) | NCT01192464 | Active, not recruiting | HL, NHL | autologous CAR.CD30 EBV specific-CTLs | Baylor College of Medicine |
| [Study of CAR-T Cells Expressing CD30 and CCR4 for r/r CD30+ HL and CTCL](https://clinicaltrials.gov/study/NCT03602157?intr=CD30%20CAR&page=1&limit=50&rank=32) | NCT03602157 | Active, not recruiting | Lymphoma, Immune System Diseases, ID, 10 more | ATLCAR.CD30.CCR4 cells, ALTCAR.CD30 cells, Bendamustine, 1 more | UNC Lineberger Comprehensive Cancer Center |
| [ATLCAR.CD30.CCR4 for CD30+ HL ATLCAR.CD30.CCR4 Cells](https://clinicaltrials.gov/study/NCT06090864?intr=CD30%20CAR&page=1&limit=50&rank=33) | NCT06090864 | Recruiting | r/r HL | Chemotherapy, Cell infusion | UNC Lineberger Comprehensive Cancer Center |
| [Administration of T Lymphocytes for Hodgkin's Lymphoma and Non-Hodgkin's Lymphoma (CART CD30)](https://clinicaltrials.gov/study/NCT01316146?intr=CD30%20CAR&page=1&limit=50&rank=34) | NCT01316146 | Withdrawn | NHL, HL | CAR.CD30 T cells | UNC Lineberger Comprehensive Cancer Center |
| [Compassionate Use Re-Infusion of ATLCAR.CD30](https://clinicaltrials.gov/study/NCT03914885?intr=CD30%20CAR&page=1&limit=50&rank=35) | NCT03914885 | No longer available | HL, Adult | ATLCAR.CD30, Bendamustine, Fludarabine | UNC Lineberger Comprehensive Cancer Center |
| [A Study of LCAR-HL30 in Subjects With Relapsed/Refractory Hodgkin's Lymphoma and Anaplastic Large Cell Lymphoma](https://clinicaltrials.gov/study/NCT06494371?intr=CD30%20CAR&page=1&limit=50&rank=36) | NCT06494371 | Recruiting | HL, ALCL | LCAR-HL30 cells | Ruijin Hospital |

ALCL: Anaplastic Large Cell Lymphoma; HL: Hodgkin Lymphoma; NHL: Non-Hodgkin Lymphoma; r/r: relapsed/refractory; LCA: Large Cell Anaplastic; HD: Hodgkin Disease; DLBCL: Diffuse Large B Cell Lymphoma; PTCL: Peripheral T Cell Lymphoma; ENK/TCL: Extranodal Natural Killer/T-Cell Lymphoma; cHL: Classical Hodgkin Lymphoma; ID: Immunoproliferative Disorders; ISD: Immune System Diseases; Interventions are reported as indicated on the website <https://clinicaltrials.gov/>

Supplementary Table S2. List of some Clinical Trials with HER2-directed CAR immune cells in different solid tumors.

| **Study Title** | **NCT Number** | **Status** | **Conditions** | **Interventions** | **Sponsor** |
| --- | --- | --- | --- | --- | --- |
| [Personalized Chimeric Antigen Receptor T Cell Immunotherapy for Patients With Recurrent Malignant Gliomas](https://clinicaltrials.gov/study/NCT03423992?intr=Her2%20Chimeric%20antigen%20receptor&page=1&viewType=Table&rank=1) | NCT03423992 | Unknown status | Glioma, GBM, Recurrence Tumor | EGFRvIII, IL13Rα2, Her-2, CD133, EphA2, GD2 CAR T cells | Xuanwu Hospital, Beijing |
| [Evaluate the Safety and Efficacy of CAR-T in the Treatment of Pancreatic Cancer.](https://clinicaltrials.gov/study/NCT03267173?intr=Her2%20Chimeric%20antigen%20receptor&page=1&viewType=Table&rank=2) | NCT03267173 | Unknown status | PC, CAR | Mesothelin/PSCA/CEA/HER2/MUC1/, EGFRvIII and other CAR T cell | First Affiliated Hospital of Harbin Medical University |
| [HER2-specific CAR T Cell Locoregional Immunotherapy for HER2-positive Recurrent/Refractory Pediatric CNS Tumors](https://clinicaltrials.gov/study/NCT03500991?intr=Her2%20Chimeric%20antigen%20receptor&page=1&viewType=Table&rank=3) | NCT03500991 | Active, not recruiting | CNS Tumor, Pediatric Glioma, Ependymoma, 6 more | HER2 or EGFR CAR T cell | Seattle Children's Hospital |
| [HER2-CAR T Cells in Treating Patients With Recurrent Brain or Leptomeningeal Metastases](https://clinicaltrials.gov/study/NCT03696030?intr=Her2%20Chimeric%20antigen%20receptor&page=1&viewType=Table&rank=4) | NCT03696030 | Active, not recruiting | Malignant Neoplasm, Metastatic Malignant Neoplasm in the Brain or in the Leptomeninges, 2 more | HER2 CAR T-Cell Therapy | City of Hope Medical Center |
| [Safety and Activity Study of HER2-Targeted Dual Switch CAR-T Cells (BPX-603) in Subjects With HER2-Positive Solid Tumors](https://clinicaltrials.gov/study/NCT04650451?intr=Her2%20Chimeric%20antigen%20receptor&page=1&viewType=Table&rank=5) | NCT04650451 | Suspended | HER-2 amplified, HER2+ GC, HER2+ BC, 2 more | HER2 CAR T cell therapy | Bellicum Pharmaceuticals |
| [Chimeric Antigen Receptor-Modified T Cells for Breast Cancer](https://clinicaltrials.gov/study/NCT02547961?intr=Her2%20Chimeric%20antigen%20receptor&page=1&viewType=Table&rank=6) | NCT02547961 | Withdrawn | BC | HER-2-targeting CAR T cells infusion | Fuda Cancer Hospital, Guangzhou |
| [HER2-specific Chimeric Antigen Receptor (CAR) T Cells for Children With Ependymoma](https://clinicaltrials.gov/study/NCT04903080?intr=Her2%20Chimeric%20antigen%20receptor&page=1&viewType=Table&rank=7) | NCT04903080 | Active, not recruiting | Ependymoma | HER2 Specific CAR T cell | Pediatric Brain Tumor Consortium |
| [T Cells Expressing HER2-specific Chimeric Antigen Receptors(CAR) for Patients With HER2-Positive CNS Tumors](https://clinicaltrials.gov/study/NCT02442297?intr=Her2%20Chimeric%20antigen%20receptor&page=1&viewType=Table&rank=8) | NCT02442297 | Active, not recruiting | r/r Brain Tumor | HER2-specific T cells | Baylor College of Medicine |
| [Treatment of Chemotherapy Refractory Human Epidermal Growth Factor Receptor-2( HER-2) Positive Advanced Solid Tumors](https://clinicaltrials.gov/study/NCT01935843?intr=Her2%20Chimeric%20antigen%20receptor&page=1&viewType=Table&rank=9) | NCT01935843 | Unknown status | Advanced HER2+ Solid Tumors refractory to Chemotherapy HER-2 Antibody | CART-HER-2 | Chinese PLA General Hospital |
| [Her2 Chimeric Antigen Receptor Expressing T Cells in Advanced Sarcoma](https://clinicaltrials.gov/study/NCT00902044?intr=Her2%20Chimeric%20antigen%20receptor&page=1&viewType=Table&rank=10) | NCT00902044 | Active, not recruiting | Sarcoma | Autologous HER2 CAR T cells, fludarabine, cyclophosphamide, 1 more | Baylor College of Medicine |
| [A Clinical Study on the Safety and Efficacy of Chimeric Antigen Receptor T-cell (CART) in the Treatment of Solid Tumors](https://clinicaltrials.gov/study/NCT05745454?intr=Her2%20Chimeric%20antigen%20receptor&page=2&viewType=Table&rank=11) | NCT05745454 | Not yet recruiting | Solid Tumor | HER2-E-CART cells | su haichuan |
| [Human HER2-targeted Macrophages Therapy for HER2-positive Advanced Gastric Cancer With Peritoneal Metastases](https://clinicaltrials.gov/study/NCT06224738?intr=Her2%20Chimeric%20antigen%20receptor&page=2&viewType=Table&rank=12) | NCT06224738 | Not yet recruiting | GC | human HER2-targeted CAR-M cells | First People's Hospital of Hangzhou |
| [Gene Therapy Using Anti-Her-2 Cells to Treat Metastatic Cancer](https://clinicaltrials.gov/study/NCT00924287?intr=Her2%20Chimeric%20antigen%20receptor&page=2&viewType=Table&rank=13) | NCT00924287 | Terminated  [WITH RESULTS](https://clinicaltrials.gov/study/NCT00924287?intr=Her2%20Chimeric%20antigen%20receptor&page=2&viewType=Table&tab=results&rank=13) | Metastatic Cancer | in vitro tumor reactive, HER2 CAR T gene-transduced PBL plus IV aldesleukin, cyclophosphamide, fludarabine, 1 more | National Cancer Institute (NCI) |
| [HER2 Chimeric Antigen Receptor (CAR) T Cells in Combination With Checkpoint Blockade in Patients With Advanced Sarcoma](https://clinicaltrials.gov/study/NCT04995003?intr=Her2%20Chimeric%20antigen%20receptor&page=2&viewType=Table&rank=14) | NCT04995003 | Recruiting | Sarcoma, HER-2Overexpression OS, 5 more | T cells or CAR T cells, pembrolizumab, nivolumab | Baylor College of Medicine |
| [CAR-monocytes for the Treatment of HER2 Overexpressing Solid Tumors](https://clinicaltrials.gov/study/NCT06254807?intr=Her2%20Chimeric%20antigen%20receptor&page=2&viewType=Table&rank=15) | NCT06254807 | Active, not recruiting | HER2+ | CT-0525 (HER2 monocytes) | Carisma Therapeutics Inc |
| [Sequential Infusion of CD146-Targeted and HER2-Targeted CAR T Cells in Patients With Advanced Sarcomas](https://clinicaltrials.gov/study/NCT07066982?intr=Her2%20Chimeric%20antigen%20receptor&page=2&viewType=Table&rank=16) | NCT07066982 | Recruiting | Sarcoma, KS, ES 6 more | CD146/HER2 CAR-T cells | Essen Biotech |
| [TRAIL-R2 and HER2 Bi-Specific Chimeric Antigen Receptor (CAR) T Cells for the Treatment of Metastatic Breast Cancer](https://clinicaltrials.gov/study/NCT06251544?intr=Her2%20Chimeric%20antigen%20receptor&page=2&viewType=Table&rank=17) | NCT06251544 | Not yet recruiting | BC, Breast Tumor, 5 more | HTR2 T Cells | Baylor College of Medicine |
| [A Study of CART-TnMUC1 in Patients With TnMUC1-Positive Advanced Cancers](https://clinicaltrials.gov/study/NCT04025216?intr=Her2%20Chimeric%20antigen%20receptor&page=2&viewType=Table&rank=18) | NCT04025216 | Terminated | NSCLC, OC, FTC 3 more HER2- | CART-TnMUC1, cyclophosphamide, fludarabine | Kite, A Gilead Company |
| [CMV-specific Cytotoxic T Lymphocytes Expressing CAR Targeting HER2 in Patients With GBM](https://clinicaltrials.gov/study/NCT01109095?intr=Her2%20Chimeric%20antigen%20receptor&page=2&viewType=Table&rank=19) | NCT01109095 | Completed | GBM | HER.CAR CMV-specific CTLs | Baylor College of Medicine |
| [CAR-macrophages for the Treatment of HER2 Overexpressing Solid Tumors](https://clinicaltrials.gov/study/NCT04660929?intr=Her2%20Chimeric%20antigen%20receptor&page=2&viewType=Table&rank=20) | NCT04660929 | Active, not recruiting | HER2+, Adenocarcinoma, Bile Duct Cancer, 28 more | CT-0508 (HER2 CAR-M), pembrolizumab | Carisma Therapeutics Inc |
| [Autologous huMNC2-CAR44 or huMNC2-CAR22 T Cells for Breast Cancer Targeting Cleaved Form of MUC1 (MUC1*)](https://clinicaltrials.gov/study/NCT04020575?intr=Her2%20Chimeric%20antigen%20receptor&page=3&viewType=Table&rank=21) | NCT04020575 | Recruiting | mBC | huMNC2-CAR44 CAR T cells or huMNC2-CAR22 CAR T cells, huMNC2-CAR44 CAR T cells or huMNC2-CAR22 CAR T cells @ RP2D | Minerva Biotechnologies Corporation |
| [A Phase I Trial of CCT303-406 in Patients With Relapsed or Refractory HER2 Positive Solid Tumors](https://clinicaltrials.gov/study/NCT04511871?intr=Her2%20Chimeric%20antigen%20receptor&page=3&viewType=Table&rank=22) | NCT04511871 | Active, not recruiting | Solid Tumor, GCBC, 2 more | CCT303-406 | Shanghai PerHum Therapeutics Co., Ltd. |
| [Study of MT-302 in Adults With Advanced or Metastatic Epithelial Tumors](https://clinicaltrials.gov/study/NCT05969041?intr=Her2%20Chimeric%20antigen%20receptor&page=3&viewType=Table&rank=23) | NCT05969041 | Recruiting | Epithelial Tumors, Malignant | MT-302 (A) | Myeloid Therapeutics |
| [QUILT-3.047: NANT Head and Neck Squamous Cell Carcinoma (HNSCC) Vaccine: Combination Immunotherapy in Subjects With HNSCC Who Have Progressed on or After Chemotherapy and PD-1/PD-L1 Therapy](https://clinicaltrials.gov/study/NCT03169764?intr=Her2%20Chimeric%20antigen%20receptor&page=3&viewType=Table&rank=24) | NCT03169764 | Withdrawn | HNSCC | avelumab, bevacizumab, capecitabine, 19 more | ImmunityBio, Inc. |
| [QUILT-3.050: NANT Colorectal Cancer (CRC) Vaccine: Combination Immunotherapy in Subjects With Recurrent or Metastatic CRC](https://clinicaltrials.gov/study/NCT03169777?intr=Her2%20Chimeric%20antigen%20receptor&page=3&viewType=Table&rank=25) | NCT03169777 | Withdrawn | CRC | avelumab, bevacizumab, capecitabine, 19 more | ImmunityBio, Inc. |

BC: breast cancer/carcinoma, GC: gastric carcinoma; CRC: colorectal carcinoma, HNSCC: head and neck squamous cell carcinoma; GBM: glioblastoma/glioma multiforme; NSCLC: non-small cell lung cancer; OC: ovarian cancer/carcinoma; KS: Kaposi Sarcoma; ES: Ewing Sarcoma; OS: Osteo Sarcoma; FTC: Fallopian Tube Cancer; PBL: peripheral blood lymphocyte; PC: Pancreatic Cancer; CAR-M: CAR Macrophages; Interventions are reported as indicated on the website <https://clinicaltrials.gov/>
